# Impact of symmetry-breaking of non-fullerene acceptors for efficient and stable organic solar cells†

**DOI:** 10.1039/d1sc04153c

**Published:** 2021-10-13

**Authors:** Peddaboodi Gopikrishna, Huijeong Choi, Do Hui Kim, Jun Ho Hwang, Youngwan Lee, Hyeonwoo Jung, Gyeonghwa Yu, Telugu Bhim Raju, Eunji Lee, Youngu Lee, Shinuk Cho, BongSoo Kim

**Affiliations:** Department of Chemistry, Ulsan National Institute of Science and Technology (UNIST) 50 UNIST-gil Ulsan 44919 Republic of Korea bongsoo@unist.ac.kr; Department of Physics and EHSRC, University of Ulsan 93 Daehak-ro, Nam-gu Ulsan 44610 Republic of Korea sucho@ulsan.ac.kr; School of Materials Science and Engineering, Gwangju Institute of Science and Technology (GIST) 123 Cheomdangwagi-ro, Buk-gu Gwangju 61005 Republic of Korea; Department of Energy Science and Engineering, Daegu Gyeongbuk Institute of Science and Technology (DGIST) 333, Techno Jungang-daero, Hyeonpung-eup, Dalseong-gun Daegu 42988 Republic of Korea

## Abstract

The concurrent enhancement of short-circuit current (*J*_SC_) and open-circuit voltage (*V*_OC_) is a key problem in the preparation of efficient organic solar cells (OSCs). In this paper, we report efficient and stable OSCs based on an asymmetric non-fullerene acceptor (NFA) IPC-BEH-IC2F. The NFA consists of a weak electron-donor core dithienothiophen[3,2-*b*]-pyrrolobenzothiadiazole (BEH) and two kinds of strong electron-acceptor (A) units [9*H*-indeno[1,2-*b*]pyrazine-2,3-dicarbonitrile (IPC) with a tricyclic fused system and 2-(5,6-difluoro-3-oxo-2,3-dihydro-1*H*-inden-1-ylidene)malononitrile (IC2F)]. For comparison, the symmetric NFAs IPC-BEH-IPC and IC2F-BEH-IC2F were characterised. The kind of flanking A unit significantly affects the light absorption features and electronic structures of the NFAs. The asymmetric IPC-BEH-IC2F has the highest extinction coefficient among the three NFAs owing to its strong dipole moment and highly crystalline feature. Its highest occupied molecular orbital (HOMO) and lowest unoccupied molecular orbital (LUMO) levels lie between those of the IPC-BEH-IPC and IC2F-BEH-IC2F molecules. The IPC group also promotes molecular packing through the tricyclic π-conjugated system and achieves increased crystallinity compared to that of the IC2F group. Inverted-type photovoltaic devices based on p-type polymer:NFA blends with PBDB-T and PM6 polymers as p-type polymers were fabricated. Among all these devices, the PBDB-T:IPC-BEH-IC2F blend device displayed the best photovoltaic properties because the IPC unit provides balanced electronic and morphological characteristics. More importantly, the PBDB-T:IPC-BEH-IC2F-based device exhibited the best long-term stability owing to the strongly interacting IPC moiety and the densely packed PBDB-T:IPC-BEH-IC2F film. These results demonstrate that asymmetric structural modifications of NFAs are an effective way for simultaneously improving the photovoltaic performance and stability of OSCs.

## Abstract 2

A 9*H*-indeno[1,2-*b*]pyrazine-2,3-dicarbonitrile (IPC) moiety in asymmetric non-fullerene acceptors promotes the formation of a densely packed crystalline structure, enabling efficient and long-term stable organic solar cells.

## Graphical abstract

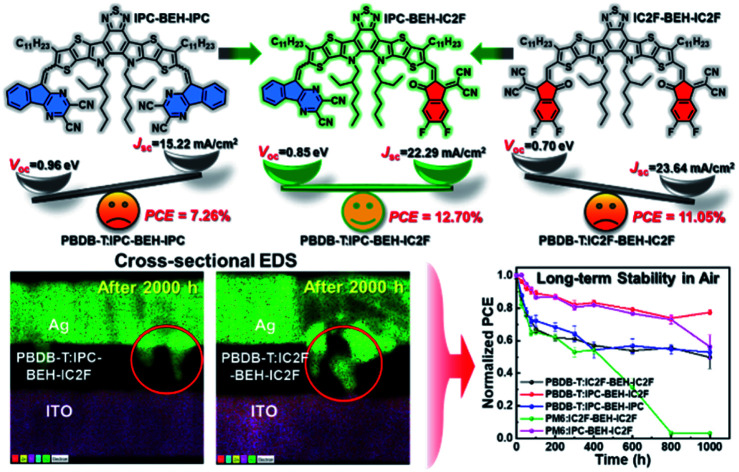

## Introduction

In the past two decades, organic solar cells (OSCs) have received considerable attention because they can be produced by a low-cost process and are suitable for lightweight, flexible, large-area, and semi-transparent applications.^1–3^ The photovoltaic properties of OSCs depend mainly on the photoactive materials.^4–6^ The applied photoactive materials are blends of p- and n-type materials with suitable ratios. To prepare an efficient OSC, the photoactive blend should exhibit visible–near-infrared light absorption, high hole and electron mobilities, and a favourable molecular orbital energy alignment between the p- and n-type materials.^7–9^ Several high-performance p-type polymers such as PBDB-T and halogen atom-containing PBDB-T derivatives (*e.g.* PM6) have been developed since non-fullerene acceptors (NFAs) have been used as n-type materials in OSCs.^10–12^ Photoactive films based on PBDB-T derivatives and NFAs have shown high power conversion efficiencies (PCEs) of over 15%.^13^ These polymers have important characteristics including complementary light absorption, a suitable alignment of their highest occupied molecular orbital (HOMO) and lowest unoccupied molecular orbital (LUMO) levels with respect to those of NFAs, and an optimal degree of phase separation for forming a suitable morphology in the photoactive blend film.

Most efficient NFAs consist of a fused-ring central electron donor (D) core unit and two electron accepting end units (As) with an ADA structure.^14^ J. Yuan *et al.* developed a more elaborate ADADA-type core structure IC2F-BEH-IC2F (originally called “Y6”) NFA. They inserted an electron-deficient benzo[*c*]1,2,5-thiadiazole (BT) moiety into the central core. This change reduces the electron donating strength, and the energy levels of its frontier orbitals are well matched with those of the p-type PM6 polymer, which results in impressive photovoltaic properties.^15^ Moreover, the substituted alkyl chains or A units in IC2F-BEH-IC2F were modified and the heteroatoms were replaced to improve the photovoltaic properties.^16–21^ Despite the rapid progress in the improvement of the PCEs of photovoltaic devices based on IC2F-BEH-IC2F derivatives, the trade-off between the short-circuit current density (*J*_SC_) and open-circuit voltage (*V*_OC_) remains a problem. For instance, the modification of central D cores or flanked A units for improved light absorption can increase *J*_SC_. This change would be accompanied by an upshifted HOMO level or a lowered LUMO level of the NFAs. The upshifted HOMO levels of NFAs would lead to less efficient free charge generation, and the lowered LUMO levels of NFAs would result in a decreased *V*_OC_. To avoid these problems, the synthesis of an NFA with an asymmetric structure with the use of two different A units will be a simple and effective strategy. Asymmetric NFAs that are flanked with two different A units have rarely been studied.^22–26^ This approach allows the fine-tuning of the optoelectronic properties of the NFAs, which results in improved photovoltaic properties. Z. Luo *et al.* prepared an asymmetric IC2F-BEH-IC2F derivative of BTP-2F-ThCl with IC2F and ThCl acceptors.^23^ When BTP-2F-ThCl was paired with PM6, a PCE of 17.06% was achieved by the simultaneous increases in *V*_OC_ and *J*_SC_ compared to that of IC2F-BEH-IC2F molecules. The excellent performance characteristics originate from the slight increase in the molecular LUMO level through the replacement of IC2F with a weaker electron-accepting ThCl unit. T. Liu *et al.* reported the preparation of an asymmetric IC2F-BEH-IC2F derivative of SY1 with the use of a monochloro IC group in place of the IC2F group; the resulting PCE was 16.83%.^24^ The SY1 molecules exhibited an upshifted LUMO and better miscibility with PM6 polymer compared to IC2F-BEH-IC2F, thereby yielding higher *V*_OC_ and fill factor (FF) values than the PM6:IC2F-BEH-IC2F device. Furthermore, H. Chen *et al.* presented asymmetric BTIC-2Cl-γCF_3_ with a new γCF_3_ acceptor unit. These molecules can form a three-dimensional network system between adjacent molecules, which results in good charge transport pathways. Moreover, the solubility of BTIC-2Cl-γCF_3_ was improved compared to that of the symmetric counterpart IC2Cl-BEH-IC2Cl; the former allows the processing of non-halogenated solvents (toluene) and results in a PCE of 16.31%.^27^ These successful examples are limited to the simple replacement of IC2F or IC2Cl units on one side. Therefore, new asymmetric NFAs should be investigated.

The commercialization of OSCs is hindered by insufficient long-term stability. OSCs degrade over time mainly due to the morphological instability of the photoactive blend film. The photoactive blend films consist of three domains: p-type polymer domain, n-type accepter domain, and mixed domain. Obtaining an optimal degree of phase-separation between p-type and n-type materials is important to achieving high photovoltaic performance and keeping the properly phase-separated morphology is key to maintaining the initial high PCEs. Many high-performance OSCs have exhibited metastable morphologies and unstable interfaces.^28–30^ Morphological changes over time resulted in decreased *J*_SC_ and FF values; changes in the molecular orientation and the degree of phase separation impede efficient charge transport and induce charge carrier recombination. To overcome this issue, researchers have studied topics in the fields of molecular design, morphology control, and interfacial device engineering.^31–34^ For instance, J. Song *et al.* synthesised a p-type PT2 copolymer; the PT2:NFA blend-based OSCs exhibited long-term stability because PT2 copolymers create an interpenetrating fibrillar network, which remains unchanged over time.^35^ Similarly, Yang *et al.* fabricated ternary OSCs by employing an n-type N2200 polymer acceptor as a third component into the PBDB-T:ITIC blend. The ternary OSC retained 80% of the initial PCE after 1000 h of storage in air because the N2200 polymer acceptor generated a firm, interpenetrating network structure.^36^ Alternatively, the optimized photoactive blend morphology can be maintained by adding a solid additive. R. Yu *et al.* synthesised a volatile solid additive SA-1 for fabricating OSCs. The OSCs containing SA-1 exhibited good long-term storage stability for 1500 h, because SA-1 enhanced the intermolecular interaction of IT-4F molecules and the crystallinity of the photoactive layer.^37^ Moreover, the degradation of the OSCs can be affected by interfacial materials so it is critical to use a stability-improving interfacial molecule. J. Yao *et al.* improved the device stability by using aliphatic amine-functionalised perylene-diimide PDINN as an organic cathode interlayer.^38^ Although these intriguing strategies have been reported till now, in-depth degradation mechanisms of OSCs have not yet been clarified; in particular, for devices based on high-performance polymer:IC2F-BEH-IC2F derivative photoactive layers. In this regard, the long-term stability of polymer:NFA-based OSCs must be studied more thoroughly to provide a molecular design rule for excellent photovoltaic properties and long-term stability.

**Fig. 1:**
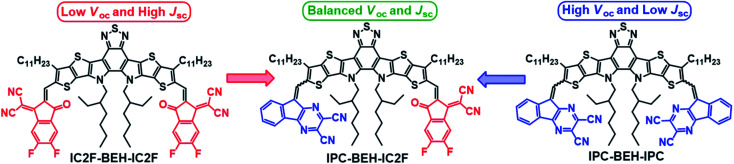
Chemical structures of IC2F-BEH-IC2F, IPC-BEH-IC2F, and IPC-BEH-IPC.

Herein, we report the synthesis and electrical characterization of a new asymmetric A_1_DADA_2_-type IPC-BEH-IC2F NFA with a new tricyclic 9*H*-indeno[1,2-*b*]pyrazine-2,3-dicarbonitrile (IPC) electron-accepting unit on one side and a new symmetric ADADA-type IPC-BEH-IPC NFA. The chemical structures of the NFA molecules used in this study are shown in Fig. 1. The designed IPC moiety contains strongly electron-withdrawing pyrazine and dicarbonitrile functional groups; its tricyclic π-conjugated structure promotes intermolecular interactions. Moreover, the nitrogen atom in the pyrazine unit can interact with sulfur in the adjacent thiophene unit of the core *via* non-covalent intramolecular interactions, which result in a planar conformation. These features facilitate strong intramolecular electronic coupling between the core and IPC unit and intermolecular packing. In addition, the optoelectronic properties of the synthesised IPC-BEH-IC2F, IPC-BEH-IPC, and IC2F-BEH-IC2F were characterised. Electronic energy levels were up-shifted and optical bandgaps were also increased in the order of IC2F-BEH-IC2F, IPC-BEH-IC2F, and IPC-BEH-IPC, which proved that IPC has slightly weaker electron-accepting properties than IC2F. Nonetheless, it is interesting to observe that the extinction coefficient of IPC-BEH-IC2F is the highest among these NFAs owing to the strong dipole moment due to its asymmetric structure. The three NFAs were blended with PM6 and PBDB-T polymers in a chlorobenzene solution containing 0.5% (v/v) 1-chloronaphthalene (CN). Among those blends, the PBDB-T:IPC-BEH-IC2F-based device has the highest PCE (12.70%), which is higher than those of its symmetric counterparts, *i.e.* PBDB-T:IC2F-BEH-IC2F (PCE = 11.05%) and PBDB-T:IPC-BEH-IPC (PCE = 7.26%). The high PCE of the PBDB-T:IPC-BEH-IC2F-based device is due to the proper balance between the miscibility and crystallinity of PBDB-T and IPC-BEH-IC2F in the blend film, compared to the characteristics of the other blend films. This aspect yields balanced *J*_SC_ and *V*_OC_ compared to those of the PBDB-T:IC2F-BEH-IC2F- and PBDB-T:IPC-BEH-IPC-based OSCs. When these NFAs are paired with PM6, all the devices exhibit poorer photovoltaic properties because of the non-ideal energy level alignment or non-ideal morphology. Moreover, the PBDB-T:IPC-BEH-IC2F device exhibits the best long-term stability under ambient conditions among all the PBDB-T:NFA or PM6:NFA devices. The characterization of the aged blend films reveals that this characteristic is strongly associated with the highly packed IPC moieties in the PBDB-T:IPC-BEH-IC2F film. In other words, a stable morphology of the photoactive blend film is achieved by enhancing the intermolecular packing in the photoactive film by using a well-controlled asymmetric NFA. Overall, this study demonstrates that the IPC unit is useful in the preparation of asymmetric NFAs; hence, the introduction of different A units into ADA-type NFAs can be an effective way for tuning the optoelectronic and photovoltaic properties of future OSCs.

## Results and discussion

### Synthesis of non-fullerene acceptor molecules

**Scheme 1:**
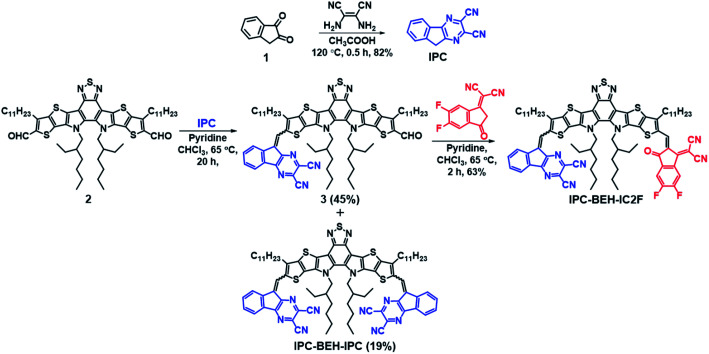
Synthetic route to IPC-BEH-IC2F and IPC-BEH-IPC.

The synthesis routes of IPC-BEH-IC2F and IPC-BEH-IPC are shown in Scheme 1; the detailed procedures are presented in the ESI.† The IPC moiety was synthesised in a single step by inducing a condensation reaction between 1*H*-indene-1,2(3*H*)-dione (**1**) and 2,3-diaminomaleonitrile. IPC-BEH-IC2F was obtained from 12,13-bis(2-ethylhexyl)-3,9-diundecyl-12,13-dihydro-1,2,5-thiadiazolo[3,4-*e*]thieno[2′′,3′′:4′,5′]thieno[2′,3′:4,5]pyrrolo[3,2-*g*]thieno[2′,3′:4,5]thieno[3,2-*b*]indole-2,10-dicarbaldehyde and IC2F *via* Knoevenagel condensation. Subsequently, its product (**3**) was reacted with IPC *via* Knoevenagel condensation. IPC-BEH-IPC was obtained during the synthesis of compound **3**. The synthesised intermediates and target NFAs were characterised with ^1^H, ^13^C, and ^19^F nuclear magnetic resonance spectra; in addition, matrix-assisted laser desorption/ionisation time-of-flight mass spectra were recorded (ESI Fig. S1–S12†). Both the synthesised NFAs exhibit good solubility in common organic solvents such as chlorobenzene (solubility = 43.3 mg mL^−1^ for IPC-BEH-IC2F and 26.7 mg mL^−1^ for IPC-BEH-IPC) and chloroform (35.7 mg mL^−1^ for IPC-BEH-IC2F and 23.5 mg mL^−1^ for IPC-BEH-IPC). Interestingly, both NFAs are more easily soluble in chlorobenzene than in chloroform because the planar IPC unit based on the tricyclic fused π-system enables stronger interactions with chlorobenzene. Note that IC2F-BEH-IC2F is more soluble in chloroform (38.7 mg mL^−1^) than in chlorobenzene (36 mg mL^−1^), and asymmetric IPC-BEH-IC2F is the most soluble among the three NFA molecules in chlorobenzene.

### Optical and electrochemical properties

**Fig. 2:**
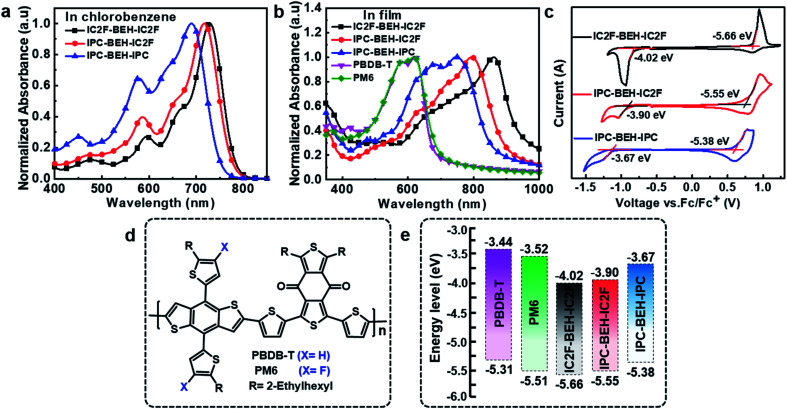
UV-vis absorption spectra of the polymers and NFAs in (a) chlorobenzene solution and (b) film. (c) Cyclic voltammograms of NFAs, (d) chemical structures of PBDB-T and PM6, and (e) energy level diagram of polymers and NFAs.

The light absorption properties of the NFAs in chlorobenzene solution and their film states were characterised by UV-visible absorption spectroscopy. In the chlorobenzene solution, the IC2F-BEH-IC2F, IPC-BEH-IC2F, and IPC-BEH-IPC molecules exhibit strong absorption at 530–780 nm with respective extinction coefficients of 1.69 × 10^5^, 1.76 × 10^5^, and 1.36 × 10^5^ M^−1^ cm^−1^ at the maximal absorption peaks (729, 716, and 690 nm), respectively (Fig. 2a and S13a†). In the thin-film state, the IC2F-BEH-IC2F, IPC-BEH-IC2F, and IPC-BEH-IPC molecules exhibit strong and broad absorption peaks at 560–980 nm with maximal absorption peaks at 861, 793, and 749 nm, respectively (Fig. 2b). The absorption maxima red-shift from 729 (*i.e.* that of the solution state) to 861 nm (by 132 nm) for IC2F-BEH-IC2F, from 716 to 793 nm (by 77 nm) for IPC-BEH-IC2F, and from 690 to 749 nm (by 59 nm) for IPC-BEH-IPC. The estimated absorption coefficients of IC2F-BEH-IC2F, IPC-BEH-IC2F, and IPC-BEH-IPC are 0.44 × 10^5^, 1.25 × 10^5^, and 0.71 × 10^5^ cm^−1^, respectively (Fig. S13b†). Note that the absorption coefficient of IPC-BEH-IC2F is much higher than those of the symmetric NFAs, which can be attributed to its strong dipole moment, highly crystalline feature and higher molecular packing in the film state (more details are discussed below). The respective absorption edges of the NFAs are 949, 895, and 856 nm, which correspond to the optical bandgaps 1.30, 1.38, and 1.44 eV, respectively. The increase in the optical bandgap reflects that the IPC moiety has a slightly weaker electron-accepting ability than the IC2F moiety. Nevertheless, the IPC-BEH-IC2F film exhibits good complementary absorption to PBDB-T and PM6 polymer films with strong absorption ability at 400–650 nm (Fig. 2b), which were used to form photoactive layers in OSCs.

The HOMO and LUMO energy levels of the NFAs in the film state were estimated by cyclic voltammetry (CV). Their cyclic voltammograms are shown in Fig. 2c. The HOMO and LUMO levels (which were determined based on the onset oxidation and reduction potentials, respectively) of IC2F-BEH-IC2F, IPC-BEH-IC2F, and IPC-BEH-IPC are −5.66/−4.02, −5.55/−3.90, and −5.38/−3.67 eV, respectively. Thus, the IPC moiety has a weaker electron withdrawing ability than the IC2F moiety. The energy levels of the NFAs and experimental results agree well with the theoretical data obtained with density functional theory (DFT) (see below). The IPC-BEH-IC2F- and IPC-BEH-IPC-based photovoltaic devices are expected to achieve higher *V*_OC_ values than IC2F-BEH-IC2F-based photovoltaic devices because of their higher LUMO energy levels (see below). Moreover, the energy levels of IPC-BEH-IC2F and IPC-BEH-IPC are more suitable for wide-bandgap p-type polymers such as PBDB-T than for PM6 (their chemical structures are shown in Fig. 2d). The energy levels of the NFAs are listed in Table 1, and the energy level diagram of the NFAs and p-type polymers used in this study are displayed in Fig. 2e.

**Table 1:** Optical and electrochemical properties of the NFAs

NFAs	*λ*_max_ a [nm]	*λ*_max_ b [nm]	HOMO^CV^ [eV]	LUMO^CV^ [eV]	E^CV^_g_ [eV]	HOMO^DFT^ [eV]	LUMO^DFT^ [eV]	E^DFT^_g_ [eV]	E^opt^_g_ [eV]
IC2F-BEH-IC2F	729	861	−5.66	−4.02	1.64	−5.61	−3.57	2.04	1.30
IPC-BEH-IC2F	716	793	−5.55	−3.90	1.65	−5.45	−3.42	2.03	1.38
IPC-BEH-IPC	690	749	−5.38	−3.67	1.71	−5.29	−3.26	2.03	1.44

a In chlorobenzene.

b In film. *E*^opt^_g_ = 1240/*λ*_onset_.

### Density functional theory (DFT) calculations

**Fig. 3:**
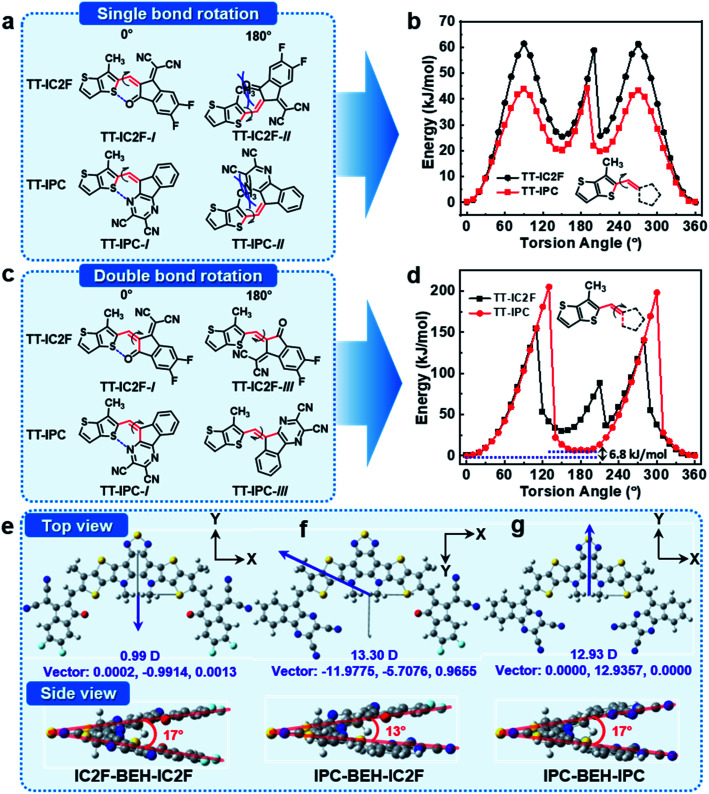
(a and c) Chemical structures of molecular conformers. (b) PES scan results for TT-IC2F and TT-IPC, where A units are rotated along the single bond. (d) PES scan results for TT-IC2F and TT-IPC, where A units are rotated along the double bond. Optimised molecular structures of NFAs: (e) IC2F-BEH-IC2F, (f) IPC-BEH-IC2F, and (g) IPC-BEH-IPC.

To investigate the molecular geometries and electronic structures of the synthesised NFAs, DFT calculations were performed at the B3LYP/6-31G(dp) level.^39^ To save calculation costs, the alkyl side chains of *n*-undecanyl and 2-ethylhexyl groups were replaced with simple methyl groups. First, we considered the configuration of the A units in the NFAs with respect to the core. The NFAs can form different structural isomers depending on the configuration of the A units; the A units can rotate along the single bond between the core and flanking A units. The relative stability of the molecular conformers of the NFAs was monitored with relaxed potential energy surface (PES) scans of model compounds of TT-IC2F and TT-IPC, each of which constitutes a partial chemical structure near the A unit (see their chemical structures in Fig. 3). Two types of PES scans were recorded by rotating the A unit with respect to the single or double bond between thienothiophene (TT) and the A unit. In the PES scan along the single bond, both TT-IC2F and TT-IPC cases indicate that TT-IC2F-*I* and TT-IPC-*I* conformers are much more stable (by 60 or 46 kJ mol^−1^, respectively) than TT-IC2F-*II* and TT-IPC-*II* conformers with 180° torsion angles (Fig. 3a and b). These results are attributed to the steric hindrance between the methyl group on the TT moiety and carbonyl moiety or between the methyl group on the TT moiety and the pyrazine moiety in the TT-IC2F-*II* and TT-IPC-*II* conformers, respectively. In addition, non-covalent intramolecular interactions of C=O⋯S or C=N⋯S at a 0° torsion angle in TT-IC2F-*I* and TT-IPC-*I* conformers can be achieved, respectively. In the PES scan along the double bond, the TT-IC2F-*I* conformer displays a lower relative energy (by 50 kJ mol^−1^) than the TT-IC2F-*III* conformer (Fig. 3c and d) at a 180° torsion angle because of steric hindrance between sulfur in the TT and the malononitrile group in IC2F. Hence, the TT-IC2F-*I* conformer is the most suitable for TT-IC2F. By contrast, the energy difference between TT-IPC-*I* and TT-IPC-*III* is very small (6.8 kJ mol^−1^), which implies that both TT-IPC-*I* and TT-IPC-*III* conformers are available. The lower energy of the TT-IPC-*I* conformer is a result of the weak non-covalent intramolecular interactions between C=N⋯S. The ratio of isomers was calculated based on the energy difference between TT-IPC-*I* and TT-IPC-*III*: Δ*E* = −*RT* ln *K* (where Δ*E* is the energy difference between the isomers, *R* the gas constant [8.315 J (K^−1^ mol^−1^)], *T* the Kelvin temperature, and *K* the equilibrium constant between the two isomers).^40^ The expected isomer ratios are 93.96% for TT-IPC-*I* and 6.04% for TT-IPC-*III*. (This theoretical estimation agrees well with the H-NMR analysis results; compound **3** contains the two conformers **3**-*I* and **3**-*III* with a ratio of 80 : 20 (Fig. S3†); IPC-BEH-IC2F contains the two conformers IPC-BEH-IC2F-*I* and IPC-BEH-IC2F-*III* with a ratio of 80 : 20 (Fig. S9†)). Since rotation along the double bond seemed to be restricted because of the huge energy barrier (200 kJ mol^−1^), both conformers have most probably been formed during the condensation reaction between compound **2** and IPC. The intermediates of the condensation reaction must be formed by the attack of the anionic IPC unit with two intermolecular cofacial π–π interactions. Subsequently, the two TT-IPC-*I* and TT-IPC-*III* conformers are produced with a higher amount of the former; this is likely because the TT-IPC-*I*-like transition state experiences less repulsion between the TT and IPC moieties. The proposed reaction mechanism is shown in Fig. S14.† Because of the small relative energy difference between TT-IPC-*I* and TT-IPC-*III*, two conformers (IPC-BEH-IC2F-*I* and IPC-BEH-IC2F-*III*) were obtained for IPC-BEH-IC2F, and three conformers (IPC-BEH-IPC-*I*, IPC-BEH-IPC-*I*&*III*, and IPC-BEH-IPC-*III*) were obtained for IPC-BEH-IPC (Fig. S15†). According to the PES scans, it can be concluded that among all the conformers, IPC-BEH-IC2F-*I* and IPC-BEH-IPC-*I* are preferred for the IPC-BEH-IC2F and IPC-BEH-IPC molecules.

In the next step, we considered the molecular geometries and electronic structures of the IC2F-BEH-IC2F, IPC-BEH-IC2F, and IPC-BEH-IPC molecules with the most preferred A unit configurations. All the optimised NFAs display almost planar geometries (Fig. 3). The torsional angles between the central core and A units of all the NFAs are small (0.54° and 0.55° for IC2F-BEH-IC2F, 0.07° and 0.27° for IPC-BEH-IC2F, and 1.88° and 1.88° for IPC-BEH-IPC, respectively). By contrast, the molecular core unit is twisted; the twist angles between the two planes containing A units (Fig. 3e, g, and f) are 17°, 13°, and 17° for IC2F-BEH-IC2F, IPC-BEH-IC2F, and IPC-BEH-IPC, respectively, thereby suggesting that the asymmetric design can promote the intermolecular packing of NFAs. The permanent dipole moments (*μ*) are 13.30 D for IPC-BEH-IC2F and 12.93 D for IPC-BEH-IPC, which are much larger than that (0.99 D) of IC2F-BEH-IC2F. The dipole moment of asymmetric IPC-BEH-IC2F is higher than that of its symmetric NFAs (*i.e.* IC2F-BEH-IC2F and IPC-BEH-IPC) because of its two different electron-accepting A units. For IC2F-BEH-IC2F and IPC-BEH-IPC, despite their symmetrical structures, IPC-BEH-IPC has a larger dipole moment than IC2F-BEH-IC2F because the strong electron-withdrawing cyano groups of the IPC unit lie in one direction, whereas the electron-withdrawing groups of the carbonyl, two cyano, and two fluorine atoms of the IC2F lie in various directions. Note that the greater dipole moments of the NFAs facilitate intermolecular packing and exciton separation.^41^ The surface plots of their frontier orbitals are shown in Fig. S16.† The electron distributions in the HOMO and LUMO levels are mainly located on the central core and A units, respectively, which promotes intramolecular charge transfer (ICT) from the central core to the A units. The calculated HOMO/LUMO energy levels of IC2F-BEH-IC2F, IPC-BEH-IC2F, and IPC-BEH-IPC are −5.61/−3.57, −5.45/−3.42, and −5.29/−3.26 eV, respectively (Table 1). These results agree well with the experimental ones and indicate the weaker electron-accepting ability of IPC compared with that of IC2F.

### Thermal properties of non-fullerene acceptors

**Fig. 4:**
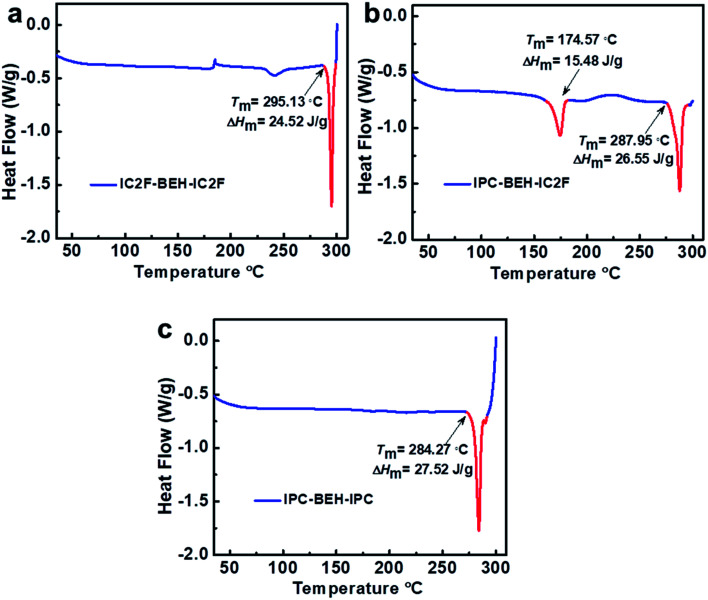
DSC curves of (a) IC2F-BEH-IC2F, (b) IPC-BEH-IC2F, and (c) IPC-BEH-IPC.

Differential scanning calorimetry (DSC) experiments were conducted to examine the molecular aggregation and crystalline characteristics of the NFAs. The DSC results reveal that the thermal transitions of NFAs depend strongly on the kind of A unit although they have identical central fused ring cores (Fig. 4). Upon heating, IC2F-BEH-IC2F and IPC-BEH-IPC each display a melting transition peak (*T*_m_) at 295.13 and 284.27 °C with a melting enthalpy (Δ*H*_m_) of 24.52 and 27.52 J g^−1^, respectively. By contrast, IPC-BEH-IC2F has two *T*_m_ peaks at 174.57 and 287.95 °C with 15.48 and 26.55 J g^−1^ Δ*H*_m_, respectively. These two melting peaks reflect two kinds of structural forms in the solid state, which are a result of its asymmetric chemical structure and the co-existence of two conformers. Note that compared to IC2F-BEH-IC2F, the IPC-BEH-IC2F and IPC-BEH-IPC molecules have higher melting enthalpies; hence, the IPC unit promotes the formation of crystalline domains in the solid state.

### Photovoltaic properties

**Fig. 5:**
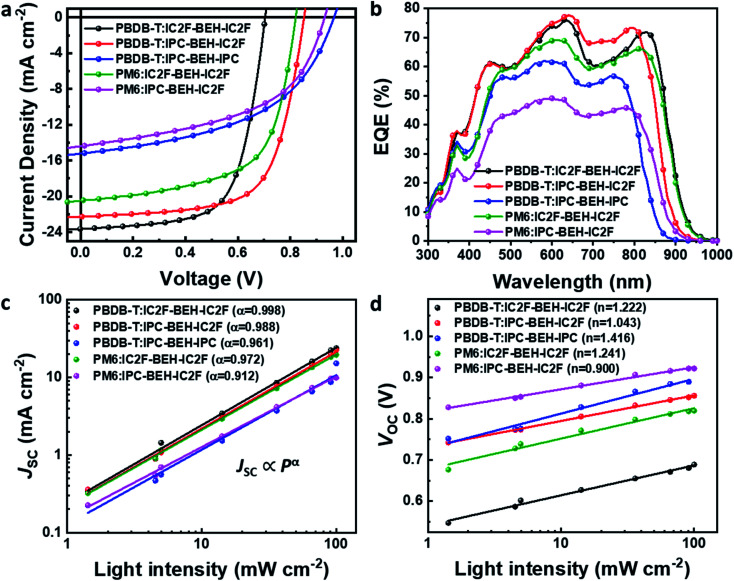
(a) *J*–*V* curves and (b) EQE spectra of PBDB-T:NFA and PM6:NFA-based OSCs. (c) Plot of *J*_SC_*vs.* light intensity and (d) *V*_OC_*vs.* light intensity for the OSCs.

The photovoltaic performance characteristics of the NFAs were characterised by fabricating inverted-type OSCs with the configuration ITO/ZnO/photoactive layer/V_2_O_5_/Ag. Commercially available PBDB-T and PM6 polymers were used as p-type polymers because of their intense absorption bands at 300–700 nm, which complement the light absorption characteristics of the NFAs. The optimal conditions for the device preparation are as follows: a polymer:NFA weight ratio of 1 : 1, the addition of 0.5 vol% 1-chloronaphthalene (CN) to the chlorobenzene solution, and pre-annealing at 130 °C for 10 min inside a glove box. We note that a higher boiling point of chlorobenzene provides more reproducible OSC results than chloroform that has been commonly used for the fabrication of IC2F-BEH-IC2F-based OSCs. In addition, unlike IC2F-BEH-IC2F, IPC-BEH-IC2F is more soluble in chlorobenzene than in chloroform, as mentioned above. The current density–voltage (*J*–*V*) characteristics of the fabricated OSCs of each blend are shown in Fig. 5, and the corresponding photovoltaic parameters are summarised in Table 2. The PBDB-T:IPC-BEH-IC2F-based device exhibits the highest PCE (12.70%) among the polymer:NFA blends in this study with a *V*_OC_ of 0.85 V, a *J*_SC_ of 22.29 mA cm^−2^, and a FF of 66.83%. The PBDB-T:IPC-BEH-IPC-based device shows a very low PCE of 7.26% with a *V*_OC_ of 0.96 V, a *J*_SC_ of 15.22 mA cm^−2^, and a FF of 49.37%. PBDB-T:IC2F-BEH-IC2F-based OSCs were fabricated under the same conditions to compare their characteristics; they exhibit a PCE of 11.05% with a *V*_OC_ of 0.70 V, a *J*_SC_ of 23.64 mA cm^−2^, and a FF of 66.38%. Compared with that of the PBDB-T:IC2F-BEH-IC2F device, the PBDB-T:IPC-BEH-IC2F device has a higher *V*_OC_ owing to the higher LUMO level of IPC-BEH-IC2F. The PBDB-T:IPC-BEH-IC2F device has a slightly lower *J*_SC_ value than the PBDB-T:IC2F-BEH-IC2F device owing to the slightly narrower absorption band of IPC-BEH-IC2F compared to that of IC2F-BEH-IC2F. Moreover, the PCE of the PBDB-T:IPC-BEH-IC2F device is increased because of the greater improvement in *V*_OC_ and the better FF although *J*_SC_ has slightly decreased. Compared with that of the PBDB-T:IPC-BEH-IPC device, the PBDB-T:IPC-BEH-IC2F device displays a higher *J*_SC_, which is attributed to the wider light absorption band of IPC-BEH-IC2F compared to that of IPC-BEH-IPC. More importantly, the PBDB-T:IPC-BEH-IPC device shows a decrease in *J*_SC_ and FF owing to the too low HOMO–HOMO offset (ΔHOMO = 0.07 eV) between PBDB-T and IPC-BEH-IPC although it has a very high *V*_OC_ (0.96 V) owing to the great upshift of the LUMO level. Therefore, when the PBDB-T polymer was blended with the asymmetric IPC-BEH-IC2F, a balance between *V*_OC_ and *J*_SC_ was achieved, which has resulted in the best PCE. In the case of the PM6-based blend films, IC2F-BEH-IC2F has better performance characteristics than IPC-BEH-IC2F because the latter has a too small HOMO energy offset (ΔHOMO = 0.04 eV) with PM6. This result demonstrates the importance of the energy level alignment between a polymer and an NFA for obtaining OSCs with excellent photovoltaic characteristics. In addition, the external quantum efficiencies (EQEs) were measured; their spectra are shown in Fig. 5b. All devices exhibit broad and intense photoresponse spectra at 300–950 nm, which agree well with the light absorption characteristics of their corresponding blend films (Fig. S17†). Both the polymer donors and NFAs contribute to the photocurrent. The *J*_SC_ values of all OSCs, integrated from EQE spectra, are consistent with the *J*_SC_ values obtained from the *J*–*V* curves.

**Table 2:** Photovoltaic parameters of the polymer:NFA blend films

Blend	*V*_OC_ (V)	*J*_SC_ (mA cm^−2^)	*J*_SC_,_EQE_ (mA cm^−2^)	FF (%)	PCE (%)
PBDB-T:IC2F-BEH-IC2F	0.704 (0.700 ± 0.004)	23.64 (22.79 ± 0.53)	22.29	66.38 (64.48 ± 2.31)	11.05 (10.29 ± 0.56)
PBDB-T:IPC-BEH-IC2F	0.852 (0.852 ± 0.004)	22.29 (21.88 ± 0.23)	20.91	66.83 (64.10 ± 1.80)	12.70 (12.01 ± 0.36)
PBDB-T:IPC-BEH-IPC	0.967 (0.968 ± 0.003)	15.22 (15.08 ± 0.21)	15.59	49.37 (48.13 ± 0.72)	7.26 (7.03 ± 0.18)
PM6:IC2F-BEH-IC2F	0.821 (0.827 ± 0.006)	20.49 (19.58 ± 0.57)	19.88	62.45 (61.22 ± 1.77)	10.50 (9.91 ± 0.47)
PM6:IPC-BEH-IC2F	0.931 (0.938 ± 0.006)	14.41 (14.13 ± 0.18)	13.30	50.45 (49.58 ± 0.14)	6.77 (6.57 ± 0.14)

The charge recombination characteristics of the PBDB-T:NFA- and PM6:NFA-based devices were investigated based on the relationship between the *J*_SC_ and light intensity (*P*): *J*_SC_ ∝ *P*^*α*^. When *α* is close to 1, bimolecular recombination has a minimal impact on the blend. As shown in Fig. 5c, the *α* values of the PBDB-T:IC2F-BEH-IC2F, PBDB-T:IPC-BEH-IC2F, PBDB-T:IPC-BEH-IPC, PM6:IC2F-BEH-IC2F, and PM6:IPC-BEH-IC2F-based OSCs are 0.998, 0.988, 0.961, 0.972, and 0.912, respectively. Hence, bimolecular recombination has a weak effect on the PBDB-T:IC2F-BEH-IC2F and PBDB-T:IPC-BEH-IC2F devices under specific *J*_SC_ conditions. In addition, we examined the dependence of *V*_OC_ on *P* based on the following relationship: 
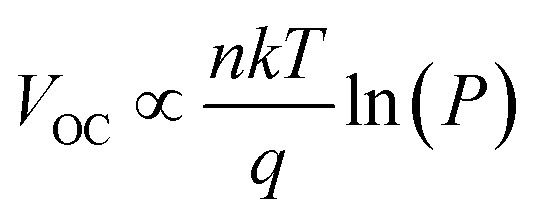
 (where *k* is the Boltzmann constant, *T* the Kelvin temperature, and *q* the elementary charge).^25^ If the slope approaches *kT*/*q* (*n* = 1), bimolecular recombination is the dominant process, whereas if it approaches *kT*/*q* (*n* > 1), monomolecular and bimolecular recombinations occur in the devices.^42,43^ According to this analysis, monomolecular recombination in the PBDB-T:IPC-BEH-IC2F- (*n* = 1.043) and PM6:IPC-BEH-IC2F (*n* = 0.900)-based devices is negligible. The slopes indicate that these two devices are only affected by bimolecular recombination under specific *V*_OC_ conditions. However, the slopes show that monomolecular and bimolecular recombinations occur in the PBDB-T:IC2F-BEH-IC2F- (*n* = 1.222), PBDB-T:IPC-BEH-IPC- (*n* = 1.416), and PM6:IC2F-BEH-IC2F (*n* = 1.241)-based devices (Fig. 5d). These results demonstrate that the PBDB-T:IPC-BEH-IC2F-based device is advantageous to obtain a high PCE because weak bimolecular and monomolecular recombinations lead to a high *J*_SC_ and FF.

Furthermore, the charge transport properties of the blend films were estimated based on space-charge limited current (SCLC) measurements; the results are shown in Fig. S18 and Table S1.† Hole-only and electron-only devices were fabricated with the configuration ITO/PEDOT:PSS/photoactive layer/MoO_3_/Ag and ITO/PEIE/photoactive layer/Cs_2_CO_3_/Al, respectively. The hole and electron mobilities of the blend films were estimated to be 5.19 × 10^−4^ and 7.33 × 10^−6^ cm^2^ V^−1^ s^−1^ for PBDB-T:IC2F-BEH-IC2F, 2.20 × 10^−4^ and 3.27 × 10^−5^ cm^2^ V^−1^ s^−1^ for PBDB-T:IPC-BEH-IC2F, 2.57 × 10^−4^ and 4.94 × 10^−5^ cm^2^ V^−1^ s^−1^ for PBDB-T:IPC-BEH-IPC, 2.82 × 10^−4^ and 1.81 × 10^−5^ cm^2^ V^−1^ s^−1^ for PM6:IC2F-BEH-IC2F, and 3.22 × 10^−4^ and 3.13 × 10^−5^ cm^2^ V^−1^ s^−1^ for PM6:IPC-BEH-IC2F, respectively. These hole mobilities are sufficiently high, while the electron mobilities are rather low. The electron mobilities in the IPC-BEH-IC2F- and IPC-BEH-IPC-based blend films are better than those in the IC2F-BEH-IC2F-based blend films. The higher crystallinity of IPC-BEH-IC2F and IPC-BEH-IPC may be the reason for the improved electron mobility (further discussion of the morphology characterization of the blends is given below).

### Blend morphology (AFM, TEM and GIWAXS study)

**Fig. 6:**
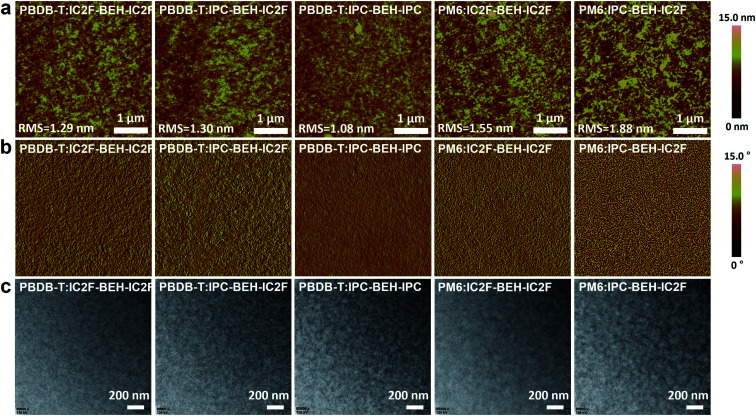
(a) AFM height images, (b) AFM phase images, and (c) TEM images of the polymer:NFA blend films.

To study more thoroughly the surface morphologies of the photoactive layers, atomic force microscopy (AFM) imaging was performed. The polymer:NFA blend films were spin-coated under optimised conditions. In Fig. 6a and b, the PBDB-T-based blend films have uniform and smooth surfaces; the RMS values are 1.29, 1.30, and 1.08 nm for PBDB-T:IC2F-BEH-IC2F, PBDB-T:IPC-BEH-IC2F, and PBDB-T:IPC-BEH-IPC, respectively. The PBDB-T:IC2F-BEH-IC2F and PBDB-T:IPC-BEH-IC2F films (both have high *J*_SC_ and FF values) exhibit similar homogeneous, nanoscopic fibrillar structures according to the height and phase images. However, the PBDB-T:IPC-BEH-IPC film has an even smoother and less clear fibrillar structure with some aggregates; this can be another reason why this blend has a low PCE. By contrast, the PM6-based blend films have more aggregated and irregular film morphologies with higher RMS values (1.55 and 1.88 nm for PM6:IC2F-BEH-IC2F and PM6:IPC-BEH-IC2F, respectively). These features are responsible for the lower device performance of the PM6-based OSC devices. In addition, to provide more in-depth insight into the blend morphologies, transmission electron microscopy (TEM) imaging was performed. Fig. 6c shows different degrees of nanoscale-phase separation depending on the employed materials. It seems that the PBDB-T-based blend films are more miscible with NFAs than the PM6-based blend films. With an increasing number of IPC moieties, the blend films show more phase separations, which is presumably because IPC moieties promote stacking more than IC2F moieties. The degree of phase separation determines the *J*_SC_ and FF values; the blend films with smaller nanoscopic phase separations have higher values, whereas the blend films with larger phase separations have lower values. Therefore, the morphology results clearly indicate that optimal intermolecular interactions and phase separations can result in high *J*_SC_ and FF values. In addition, p-type polymers and NFAs must be suitably combined to obtain a high PCE.

**Fig. 7:**
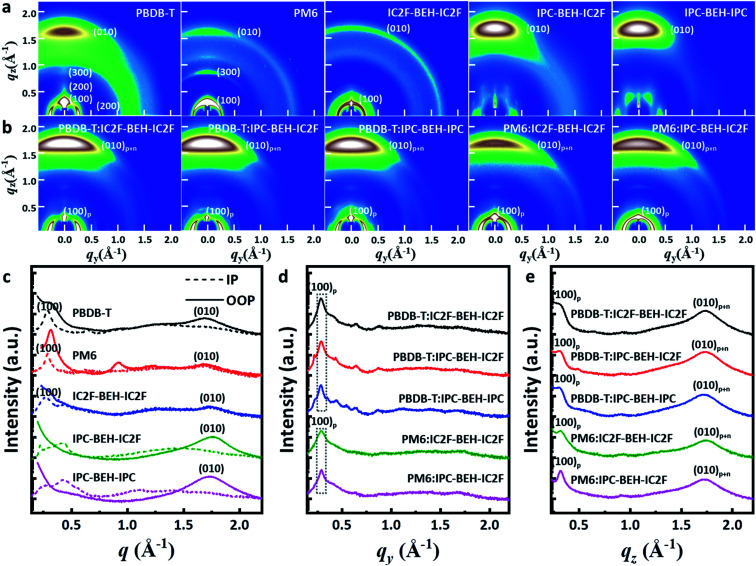
(a) GIWAXS diffraction patterns and (c) the corresponding line-cut profiles of neat films of polymers and NFAs. (b) GIWAXS diffraction patterns and (d and e) the corresponding line-cut profiles of polymer:NFA blend films in the in-plane (IP) plane and in the out-of-plane (OOP).

Two-dimensional grazing incidence wide-angle X-ray scattering (GIWAXS) measurements were performed on the neat and polymer:NFA blend films. The GIWAXS diffraction patterns and corresponding line-cut profiles of the neat and blend films are shown in Fig. 7; the fitting results are listed in Tables S2 and S3.† As shown in Fig. 7a and c, a strong (100) lamellar stacking peak at *q* = 0.288 Å^−1^ (*d* = 21.826 Å) in the in-plane (IP) direction and a strong (010) π–π stacking peak at *q* = 1.701 Å^−1^ (*d* = 3.695 Å) in the out-of-plane (OOP) direction were observed in the PBDB-T neat film. Thus, the PBDB-T polymer chains have mainly face-on orientation. In addition, higher order (200) and (300) lamellar peaks with low intensity were observed, which reflect that the PBDB-T chains form well-aligned crystal domains in the thin-neat-film state. The PM6 neat film has a strong (100) lamellar peak at *q* = 0.287 Å^−1^ (*d* = 21.898 Å) in the IP direction and a weak (010) π–π stacking peak at *q* = 1.694 Å^−1^ (*d* = 3.709 Å) in the OOP direction. Different from the PBDB-T film, the (100) peak with high intensity also occurs in the radial distribution in the OOP direction owing to cumulative disorder due to lattice defects or distortion.^44^ This suggests that the PM6 polymer chains are more randomly arranged than PBDB-T. In the case of the neat IC2F-BEH-IC2F film, the GIWAXS diffraction pattern is shaped like a half-ring, *i.e.* it has no preferred directional order with a (100) lamellar stacking peak at *q* = 0.269 Å^−1^ (*d* = 23.369 Å) in the IP plane and a weak (010) π–π stacking peak at *q* = 1.760 Å^−1^ (*d* = 3.570 Å) in the OOP direction. These features indicate that the IC2F-BEH-IC2F molecules have random molecular orientation with low crystallinity. By contrast, both IPC-BEH-IC2F and IPC-BEH-IPC molecules containing IPC moieties display face-on orientation with strong (010) π–π stacking peaks at *q* = 1.757 Å^−1^ (*d* = 3.576 Å) and 1.727 Å^−1^ (*d* = 3.638 Å) in the OOP direction, respectively. Moreover, the more IPC moieties are in the molecule, the more crystalline features appear, which indicates that IPC moieties with larger π-conjugation areas can promote intermolecular stacking. It should be also noted that strong π–π stacking is an advantageous orientation for charge transport.

All the polymer:NFA blend films exhibit a strong (100) peak in the IP direction and a weak (100) peak in the OOP direction, which mainly originate from the lamellar stacking structure of the polymer ((100)_p_). They also have a strong (010) peak in the OOP direction (denoted as (010)_p+*n*_) owing to π–π stacking interactions both between the polymers and between the NFA molecules (Fig. 7b, d, and e). In the case of the PBDB-T:NFA blend films, the (100) peaks of the PBDB-T:IC2F-BEH-IC2F, PBDB-T:IPC-BEH-IC2F, and PBDB-T:IPC-BEH-IPC films are at *q* = 0.286 Å^−1^ (*d* = 21.965 Å), 0.287 Å^−1^ (*d* = 21.864 Å), and 0.284 Å^−1^ (*d* = 22.137 Å) in the IP direction, respectively; they correspond mainly to PBDB-T polymers. According to the intensity around the (100) peak, the crystallinities are similar but slightly decreased in the following order: PBDB-T:IC2F-BEH-IC2F, PBDB-T:IPC-BEH-IC2F, and PBDB-T:IPC-BEH-IPC (Table S3†). The PBDB-T:IC2F-BEH-IC2F, PBDB-T:IPC-BEH-IC2F, and PBDB-T:IPC-BEH-IPC blend films have a strong (010) π–π stacking peak at *q* = 1.736 Å^−1^ (*d* = 3.619 Å), 1.734 Å^−1^ (*d* = 3.624 Å), and 1.721 Å^−1^ (*d* = 3.652 Å) in the OOP direction, respectively; it corresponds mainly to NFA molecular packing. According to the (010) peaks in the blend films, the (010) peak intensities of PBDB-T:IPC-BEH-IC2F and PBDB-T:IC2F-BEH-IC2F are nearly identical and slightly higher than that of the PBDB-T:IPC-BEH-IPC film (Table S3†). This agrees well with the trend of the *J*_SC_ and FF values of the devices. For the PM6:NFA blend films, the PM6:IC2F-BEH-IC2F and PM6:IPC-BEH-IC2F films have (100) lamellar stacking peaks at *q* = 0.288 Å^−1^ (*d* = 21.797 Å) and *q* = 0.289 Å^−1^ (*d* = 21.737 Å) with moderate intensities, respectively; they mainly represent PM6 polymer stacking. The (010) π–π stacking peaks of the PM6:IC2F-BEH-IC2F and PM6:IPC-BEH-IC2F films are at *q* = 1.742 Å^−1^ (*d* = 3.607 Å) and *q* = 1.727 Å^−1^ (*d* = 3.637 Å). The (010) peak intensity of PM6:IPC-BEH-IC2F is stronger than that of PM6:IC2F-BEH-IC2F because IPC-BEH-IC2F has a better molecular stacking ability than IC2F-BEH-IC2F. Therefore, the PM6:IPC-BEH-IC2F film has a higher crystallinity than the PM6:IC2F-BEH-IC2F film. Furthermore, for a given NFA, the PBDB-T:NFA blend films have a higher crystallinity than the PM6:NFA blend films. Small diffraction peaks, which are generated by NFAs, and the lamellar stacking peak of PBDB-T appear in the IP direction, while no peaks (except the lamellar peak of PM6) appear in the PM6:NFA blend films (Fig. 7d). Finally, we note that a significant structural change by blending of a polymer and an NFA occurred depending on their combination. Unlike the PM6 neat film, a significant part of the PM6 polymer exhibits face-on orientation in the PM6:NFA blend films. Simultaneously, the blending of a p-type polymer with an NFA has induced strong π–π stacking interactions between the NFA molecules, regardless of the original state of the NFA neat film. In particular, the IC2F-BEH-IC2F-based blend films exhibit highly face-on-oriented molecular stacking although the IC2F-BEH-IC2F neat state has a radial peak distribution with weak peak intensities. The promotion of π–π stacking interactions between the NFA molecules directed by the p-type polymers improves the photovoltaic performance.

**Fig. 8:**
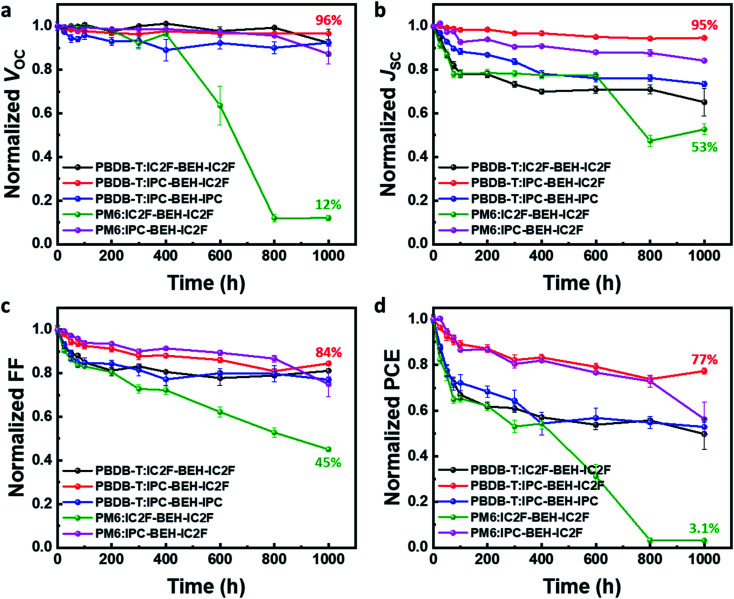
Long-term stability data of the OSC devices: (a) *V*_OC_, (b) *J*_SC_, (c) FF, and (d) PCE.

In addition to studying the photovoltaic properties directly after the device fabrication, the long-term device stability of the optimised OSCs was monitored for 1000 h in air without encapsulation; the results are summarised in Fig. 8. Among the five polymer:NFA blend devices, the PBDB-T:IPC-BEH-IC2F device displays excellent device stability; it retains over 77% of its original PCE after 1000 h. The PBDB-T:IPC-BEH-IC2F-based device retains over 95% of its *V*_OC_ and *J*_SC_ values after 1000 h, while its FF decreases gradually. Similarly, the PM6:IPC-BEH-IC2F-based device retains its initial photovoltaic properties relatively well. The PBDB-T:IPC-BEH-IPC and PBDB-T:IC2F-BEH-IC2F devices retain their initial *V*_OC_ values, whereas the *J*_SC_ and FF decrease significantly. The PM6:IC2F-BEH-IC2F device experiences the most severe device failure. Apparently, the device stability is significantly associated with the kind of NFA used in the blend films; the asymmetric IPC-BEH-IC2F is the best for long-term stability, which is followed by the symmetric IPC-BEH-IPC and then the IC2F-BEH-IC2F among the studied polymer:NFA blends. Hence, we find that the asymmetric design of NFAs and IPC moieties helps in improving the device stability.

**Fig. 9:**
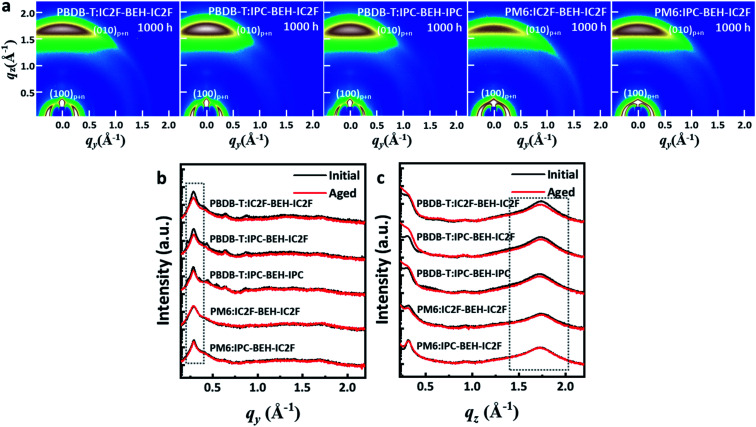
(a) GIWAXS diffraction patterns and the corresponding line-cut profiles of polymer:NFA blend films over 1000 h under ambient conditions in the dark. (b and c) Line-cut profiles of initial and 1000 h-aged polymer:NFA blend films in the in-plane (IP) and in the out-of-plane (OOP).

To study the effects of the morphological changes on the long-term device stability more thoroughly, GIWAXS measurements were performed on aged polymer:NFA blend films (they were aged under ambient conditions for 1000 h). The GIWAXS diffraction patterns and line-cut profiles of the initial and aged polymer:NFA blend films are shown in Fig. 9, and the fitting results are summarised in Table S4.† All the polymer:NFA blend films have retained their face-on orientation even after 1000 h, and the features of all blend films are similar (Fig. 9a). However, compared to the diffraction patterns of the initial state (Fig. 7), all the (100) lamellar stacking peak intensities in the IP direction and (010) π–π stacking peak intensities in the OOP direction are decreased; in addition, the minor diffraction peaks generated by the NFAs in the 0.3–0.9 range have disappeared (Fig. 9b and c). This indicates that the crystallinity of the PBDB-T:NFA blend films decreased during 1000 h of aging. Nevertheless, the crystallinity of the PBDB-T:IPC-BEH-IC2F and PBDB-T:IPC-BEH-IPC films is less degraded than that of the PBDB-T:IC2F-BEH-IC2F film. Similarly, the crystallinity of the PM6:IPC-BEH-IC2F film can be more retained than that of the PM6:IC2F-BEH-IC2F film, although the (100) and (010) peaks of the PM6:NFA blend films have decreased less than those of the PBDB-T:NFA blend films (which is presumably because the crystallinity of the PM6:NFA blend films is already quite low compared to the initial state). Therefore, these results indicate that the IPC moiety determines morphological changes because it induces stronger intermolecular interaction than the IC2F moiety, which improves the device stability (Fig. 8). However, the device stability is not simply correlated only with the change in crystallinity.

Furthermore, AFM images were taken to investigate further the surface morphological changes of the 1000 h-aged polymer:NFA blend films. Their AFM heights and phase images are shown in Fig. S19.† The PBDB-T:IC2F-BEH-IC2F and PBDB-T:IPC-BEH-IC2F blend films have similar and slightly smoother surface morphologies than those of the initial states. By contrast, the PBDB-T:IPC-BEH-IPC, PM6:IPC-BEH-IC2F, and PM6:IC2F-BEH-IC2F blend films have become rougher. According to the careful examination of the samples, the PBDB-T:IPC-BEH-IC2F blend film has retained the uniform nanoscopic fibrillar structure, whereas the PBDB-T:IC2F-BEH-IC2F and PBDB-T:IPC-BEH-IPC blend films have slightly more granular morphologies. In particular, the PM6:IC2F-BEH-IC2F blend film has a more aggregated morphology than that of the initial state. The degree of morphological change in the AFM images seems closely related to the long-term device stability.

**Fig. 10:**
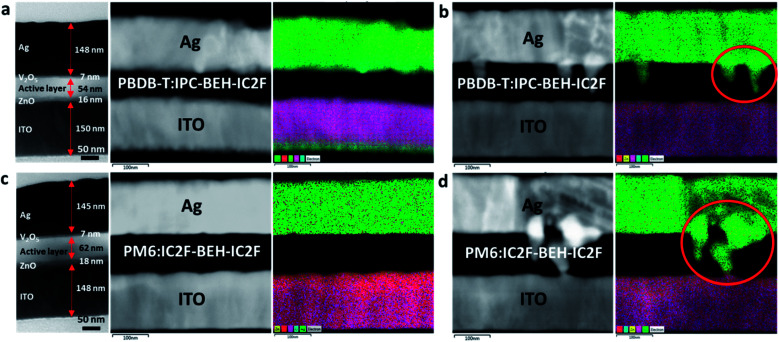
Cross-sectional TEM micrographs and layered electron dispersive spectroscopy (EDS) elemental maps for the PBDB-T:IPC-BEH-IC2F- and PM6:IC2F-BEH-IC2F-based devices before and after 2000 hours. (a and b) Fresh and aged PBDB-T:IPC-BEH-IC2F-based device. (c and d) Fresh and aged PM6:IC2F-BEH-IC2F-based device.

To gain more insight into the long-term stability characteristics of the devices, cross-sectional TEM and electron dispersive spectroscopy (EDS) images were taken. For this study, the PBDB-T:IPC-BEH-IC2F- and PM6:IC2F-BEH-IC2F-based devices were chosen, which exhibit the best and poorest long-term stability, respectively. The samples were prepared by focused ion beam milling. The cross-sectional TEM micrographs and layered EDS elemental maps of the fresh and 2000 h-aged devices are presented in Fig. 10. The fresh PBDB-T:IPC-BEH-IC2F- and PM6:IC2F-BEH-IC2F-based devices have layered structures, as expected (Fig. 10a and c). The darkest areas are the Ag electrodes (approximately 150 nm thickness), whereas the photoactive layers (approximately 60 nm) are the bright areas. The hole and electron transporting layers (7 nm V_2_O_5_ and 16 nm ZnO, respectively) between the active layer and Ag or ITO electrodes are grey. In the EDS image, the Ag electrode appears bright-grey (or green in the elemental map), the photoactive layer appears black, and the ITO appears bright-grey (or violet in the elemental map). After 2000 h of aging, both PBDB-T:IPC-BEH-IC2F- and PM6:IC2F-BEH-IC2F-based devices exhibit significant changes; Ag atoms of the Ag electrode have penetrated the photoactive layer (Fig. 10b and d); these areas are indicated by red circles in the EDS elemental map. It should be noted that the penetration of Ag atoms in the PM6:IC2F-BEH-IC2F-based device is much more severe than that in the PBDB-T:IPC-BEH-IC2F-based device. According to the GIWAXS and AFM results (Fig. 9 and S19†), (i) the PBDB-T:IPC-BEH-IC2F blend film has a high degree of crystallinity, whereas the PM6:IC2F-BEH-IC2F blend film has the lowest degree of crystallinity among the five blend films in both the initial state and the aged state; (ii) the former has retained the initial surface morphology better than the latter during aging. Therefore, we find that the degree of Ag penetration depends on the evolution of the molecular packing and film morphology of the photoactive layer during aging. Also, we note that these features are mainly attributed to the strong π–π intermolecular stacking ability of IPC moieties and the higher degree of crystallinity of the PBDB-T polymer compared to that of the PM6 polymer, which delays the Ag atom diffusion. In other words, the more crystalline PBDB-T:IPC-BEH-IC2F film has effectively resisted the diffusion of Ag atoms, whereas the less crystalline PM6:IC2F-BEH-IC2F film has allowed the diffusion of Ag through the amorphous phase or grain boundaries. These results demonstrate that the densely packed morphology of the polymer:NFA blend film is crucial for preventing the penetration of Ag atoms into the photoactive layer, which leads to good long-term device stability.

## Conclusion

In summary, we designed and synthesised two NFAs (IPC-BEH-IC2F and IPC-BEH-IPC) by introducing IPC and IC2F as electron-acceptor units. The absorption and electronic energy levels of the NFAs were well tuned; the two NFAs showed excellent molecular packing and improved crystalline characteristics compared to those of IC2F-BEH-IC2F. Interestingly, the up-shifted *E*_LUMO_ of the NFAs resulted in a higher *V*_OC_ than that of IC2F-BEH-IC2F when blended with PBDB-T and PM6. OSCs based on PBDB-T:IPC-BEH-IC2F had the highest PCE value of 12.70% owing to their high *V*_OC_ of 0.85 V and *J*_SC_ of 22.29 mA cm^−2^. In addition to the good device performance, the PBDB-T:IPC-BEH-IC2F-based device exhibited good long-term device stability. The high device performance and long-term stability of the PBDB-T:IPC-BEH-IC2F-based OSC were strongly associated with the optimal stacking ability of asymmetric IPC-BEH-IC2F according to morphological studies. Our work demonstrates that the strategy of using asymmetric NFAs in OSCs can be promising in that it allows us to tune the optoelectronic properties and degree of crystallinity of NFAs, leading to enhanced photovoltaic properties.

## Data availability

All experimental and supporting data are provided in the ESI.†

## Author contributions

P. Gopikrishna designed, synthesised, and characterised the NFAs. Also, P. Gopikrishna conducted UV-vis absorption, CV, DSC, and DFT calculations. H. Choi performed the device fabrication and characterization of OSCs. J. H. Hwang and E. Lee conducted cross-sectional TEM of OSCs. P. Gopikrishna and H. Choi wrote the manuscript. D. H. Kim and Y. Lee gave the suggestions and helped in the fabrication process. H. Jung, G. Yu, and Y. Lee conducted GIWAXS experiments and analyses. T. B. Raju gave the suggestion and helped in the synthetic process. S. Cho and B. Kim supervised the project.

## Conflicts of interest

There are no conflicts of interest.

## Supplementary Material

SC-012-D1SC04153C-s001
