# Supplementary material for: Impact of symmetry-breaking of non-fullerene acceptors for efficient and stable organic solar cells
Source: Chem Sci. 2021 Oct 13;12(42):14083–97. doi: 10.1039/d1sc04153c (PMC8565381; doi:10.1039/d1sc04153c)
Supplement: SC-012-D1SC04153C-s001 [file SC-012-D1SC04153C-s001.pdf]

Electronic Supplementary Material (ESI) for Chemical Science.
This journal is © The Royal Society of Chemistry 2021

Supplementary Information

Impact of Symmetry-Breaking of Non-Fullerene Acceptors for Efficient and Stable Organic Solar Cells

Peddaboodi Gopikrishna,[1,†] Huijeong Choi, [1,†] Do Hui Kim,[2,†] Jun Ho Hwang,[3] Youngwan Lee,[1] Hyeonwoo Jung,[4] Gyeonghwa Yu,[4] Telugu Bhim Raju,[1] Eunji Lee,[3] Youngu Lee,[4] Shinuk Cho,[2,*] and BongSoo Kim,[1,*]

[1]Department of Chemistry, Ulsan National Institute of Science and Technology (UNIST), 50 UNIST-gil, Ulsan 44919, Republic of Korea

[2]Department of Physics and EHSRC, University of Ulsan, 93 Daehak-ro, Nam-gu, Ulsan 44610, Republic of Korea

[3]School of Materials Science and Engineering, Gwangju Institute of Science and Technology (GIST), 123 Cheomdangwagi-ro, Buk-gu, Gwangju 61005, Republic of Korea

[4]Department of Energy Science and Engineering, Daegu Gyeongbuk Institute of Science and Technology (DGIST), 333, Techno Jungang-daero, Hyeonpung-eup, Dalseong-gun, Daegu, 42988, Republic of Korea

*Address correspondence to sucho@ulsan.ac.kr and bongsoo@unist.ac.kr

†P. Gopikrishna, H. Choi, and D. H. Kim contribute equally to this work.

Materials and Methods:

1*H*-indene-1,2(3*H*)-dione and 2,3-diaminomaleonitrile were purchased from the Acros Organics. 12,13-bis(2-ethylhexyl)-3,9-diundecyl-12,13-dihydro-[1,2,5]thiadiazolo[3,4-*e*]thieno[2'',3'':4',5']thieno[2',3':4,5]pyrrolo[3,2-*g*]thieno[2',3':4,5]thieno[3,2-*b*]indole-2,10-dicarbaldehyde and IC2F were purchased from the Aaron Pharmatech Ltd. All solvents were purchased from commercial sources and utilized without further purification if not mentioned.

Instrumentation:

^{1}H, ^{13}C and ^{19}F nuclear magnetic resonance (NMR) spectra were obtained by a Bruker 400 MHz FT-NMR spectrometer and Agilent 400 MHz FT-NMR spectrometer in deuterated chloroform ($CDCl_3$). The molecular mass was confirmed by Ultraflex III matrix-assisted laser desorption ionization mass spectrometer (MALDI-TOF-MS). UV-Visible absorption spectra were acquired on Agilent 8453 UV-visible Spectrophotometer from chlorobenzene solutions or thin film samples that were prepared by spin coating from chlorobenzene solution with 10 mg/mL for polymers and 20 mg/mL for NFAs. Cyclic voltammetry (CV) was carried using a CorrTest electrochemical workstation with three electrodes configuration, where platinum electrodes were used as counter and working electrodes, and Ag/AgCl as the reference electrode. The thin films of the NFAs were drop casted on the platinum working electrode from the chloroform solution. 0.1 mol L^{-1} of tetrabutylammonium hexafluorophosphate (Bu_4NPF_6) was used as supporting electrolyte in anhydrous acetonitrile and ferrocene used as external standard. HOMO and LUMO energy levels were calculated by $-(E_{ox}-E_{fc}+4.8)$ eV and $-(E_{re}-E_{fc}+4.8)$ eV, where E_{ox} is an oxidation potential of samples and an oxidation potential of ferrocene, respectively. Different scanning calorimetry (DSC) measurements were performed on a TA-Instruments DSC Q200 at a scanning rate of 10 °C for min^{-1} in N_2.

Atom force microscopy (AFM) images were recorded on a Veeco dimension AFM. Transmission electron microscopy (TEM) images were obtained by a JEM-2100 electron microscope operating at an acceleration voltage of 200 kV. Grazing-incidence wide-angle X-ray scattering (GIWAXS) measurements were conducted at PLS-II 6D UNIST-PAL beamline of Pohang Accelerator Laboratory (PAL) in Republic of Korea. The X-rays coming from the bending magnet are monochromated using Si(111) double crystals and focused at the detector position using toroidal mirror. GIWAXS patterns were recorded with a 2D CCD detector

(MX225-HS, Rayonix L.L.C., USA) and X-ray irradiation time was 60 s, which was dependent on the saturation level of detector. The sample-to-detector distance was 240 mm.

The cross-sectional samples of the organic photovoltaic devices were prepared by dual beam focused ion beam system (FIB, Helios NanoLab 600, Thermo Fisher Scientific, USA) operated at 30 kV acceleration voltage. Prior to sectioning, 500 nm thick carbon and 100 nm thick Pt protective layer were deposited on the surface of the device layer. The final sectioning of the sample was applied with conventional lift-out technique and its size was approximately 5 μm × 5 μm × 100 nm.[S1] The chemical composition of the organic photovoltaic devices was analyzed by transmission electron microscope (TEM, JEM-2100, JEOL Co., Japan) operated at 200 kV acceleration voltage equipped with energy-dispersive X-ray spectroscopy detector (EDS, X-MaxN, Oxford Instrument, UK). The TEM images were acquired with a charge-coupled device (CCD, Tengra, EMSIS GmbH, Germany) camera. The data were analyzed with imaging software RADIUS (Olympus Soft Imaging Solutions, Münster, Germany).

Device Fabrication and Electrical Measurements:

OSCs were fabricated with an inverted device configuration of ITO/ZnO/photoactive layer/V_2O_5/Ag. Patterned indium tin oxide (ITO) glass substrates were cleaned in ultrasonic bath using isopropanol, deionized water, acetone, and isopropanol consecutively for every 10 min, and dried in oven at 105 ˚C for overnight. Subsequently, the ITO substrates were treated inside an ultraviolet ozone generator for 30 min. The ZnO solution was prepared at a 2:1 volume ratio of THF and diethyl zinc solution (15 wt.% in toluene, Sigma-Aldrich). The ZnO solution was spin coated on pre-cleaned ITO substrates using the 40 μL of the precursor solution at 3500 rpm for 35 s and the coated ZnO films were annealed at 130 ˚C for 20 min. The PBDB-T:NFA or PM6:NFA layers were prepared from 15 or 20 mg/mL, respectively, in chlorobenzene containing 0.5 vol% of 1-chloronaphthalene (CN), which were stirred at 80 ˚C for 12 h. The polymer:NFA weight ratios were 1:1. The photoactive layer solutions were filtered and spin coated on the ZnO layer at 2500 rpm for 60 s. The resulting thin films were annealed at 130 ˚C for 10 min inside glove box. Then, 5.5 nm of V_2O_5 and 100 nm of Ag were deposited on the photoactive layer sequentially by thermal evaporation at a pressure of 2×10^{-5} Torr. The photoactive area of the cell was 0.12 or 0.2 cm^2. The current density-voltage (J-V) curves of OSCs were recorded with Keithley 2400 measurement source units at room temperature in air. The photocurrent was

measured under 150 W Xenon lamp using a solar simulator (K201 LAB 55, McScience Inc.). The AM 1.5G light intensity was calibrated to 100 mW cm^{-2} with a standard photovoltaic reference cell. External quantum efficiency (EQE) spectra were recorded with a spectral measurement system (K3100 IQX, McScience Inc.) equipped with a 300 W xenon arc lamp, a monochromator, and an optical chopper (MC 2000, Thorlabs, Inc., USA).

The charge carrier motilities of the blends were characterized by space-charge-limited current (SCLC) method. The hole-only and the electron-only devices were fabricated with the device configuration of ITO/PEDOT:PSS/photoactive layer/MoO_3/Ag or ITO/PEIE/Active layer/Cs_2CO_3/Al, respectively. The current density-voltage (J-V) curves of OSCs were recorded with Keithley 2400 measurement source units at room temperature in air under dark, and the carrier mobilities were extracted by using space-charge-limited current (SCLC) equation.

Experimental Section:

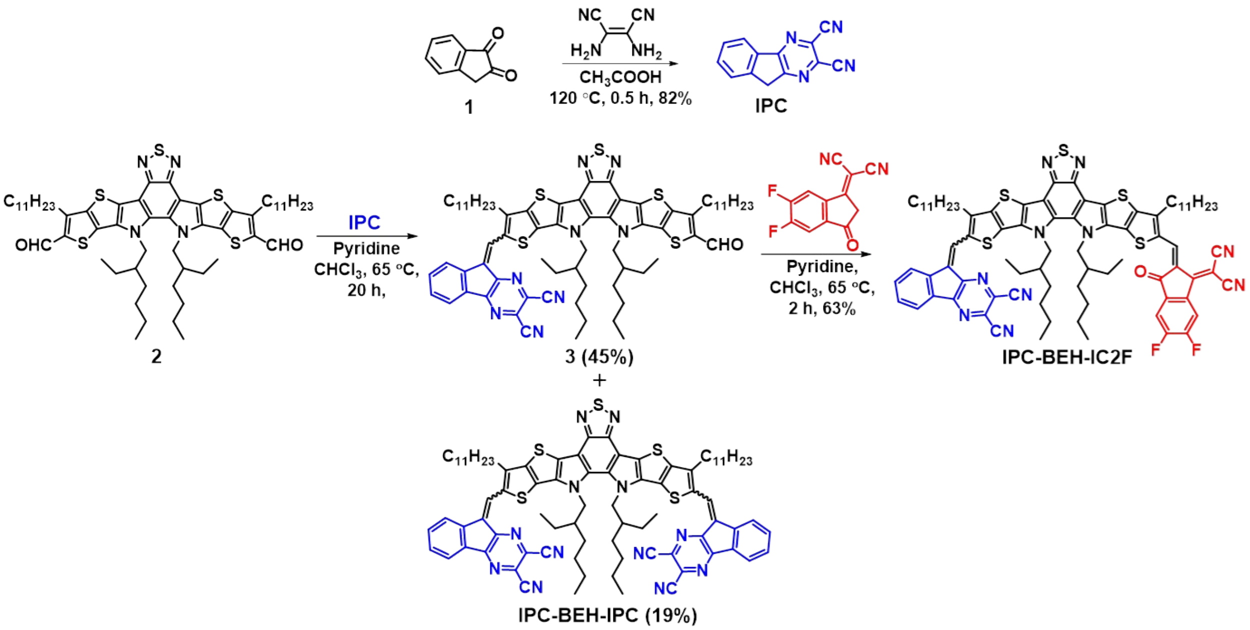

Scheme 1. Synthetic route to **IPC-BEH-IC2F** and **IPC-BEH-IPC.**

Synthetic procedure:

Synthesis of 9*H*-indeno[1,2-*b*]pyrazine-2,3-dicarbonitrile (IPC):

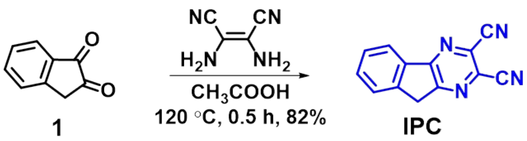

1*H*-indene-1,2(3*H*)-dione (1) (200 mg, 1.3685 mmol) and 2,3-diaminomaleonitrile (147.99 mg, 1.3685 mmol) were added into round bottom flask. Then, 15 ml of glacial acetic acid was added, and the reaction mixture stirred at 120 °C for 0.5 h under the argon atmosphere. After cooled to room temperature the reaction mixture was poured into water and the precipitate was collected by filtration. The crude product was purified by a silica gel column chromatography with dichloromethane as an eluent. A white solid of IPC was obtained (Yield: 82 %). ^{1}H NMR (400 MHz, $CDCl_3$) δ (ppm): 8.20 (d, *J*=8 Hz, 1H), 7.75-7.68 (m, 2H), 7.62 (t, 1H), 4.18 (s, 2H) ^{13}C NMR (100 MHz, $CDCl_3$) δ (ppm): 162.54, 157.47, 144.31, 135.54, 133.78, 132.63, 129.55, 129.27, ,126.23, 124.32, 114.09, 113.91, 36.53. MS (ESI): Calcd. for $C_{13}H_6N_4$ $[M]^+$: 218.0, Found: 218.0.

Synthesis of (*E*)-9-((12,13-bis(2-ethylhexyl)-10-formyl-3,9-diundecyl-12,13-dihydro-[1,2,5]thiadiazolo[3,4-*e*]thieno[2'',3'':4',5']thieno[2',3':4,5]pyrrolo[3,2-g]thieno[2',3':4,5]thieno[3,2-*b*]indol-2-yl)methylene)-9*H*-indeno[1,2-*b*]pyrazine-2,3-dicarbonitrile (3):

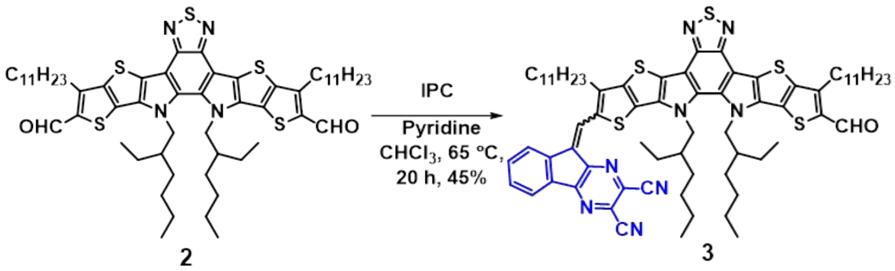

In a two-neck round-bottom flask, **2** (500 mg, 0.4868 mmol) and **IPC** (106.26 mg, 0.4868 mmol) were added, and 15 mL of anhydrous chloroform was added under the argon atmosphere. After that, pyridine (0.3 mL) was added and stirred at 65 °C for 20 h. After completion of the reaction the organic solvent was evaporated, and the crude product was purified by silica gel column chromatography with chloroform as eluent. The dark green color solid was obtained as product (Yield: 45%). ^{1}H NMR (400 MHz, $CDCl_3$) δ (ppm): 10.16 (s, 1H), 8.98 & 8.17 (d, *J*=4 & *J*=4 Hz, 1H), 8.71 & 8.31 (s, 1H), 8.27 & 7.92 (d, *J*=4 & *J*=4 Hz, 1H), 7.75 & 7.72 (t, 1H), 7.67 & 7.57 (t, 1H), 4.77 & 4.71 (d, 4H), 3.26-3.20 (m, 4H), 2.13-2.05 (m, 2H), 2.00-1.90 (m, 4H), 1.58-1.35 (m, 9H), 1.26-1.23 (m, 23H), 1.13-0.93 (m, 15H), 0.89-0.83 (m, 7H), 0.78-0.69 (m, 6H), 0.67-0.54 (m, 6H), ^{13}C NMR (100 MHz, $CDCl_3$) δ (ppm): 181.95, 154.48, 153.19, 151.60, 150.36, 147.74, 147.73, 147.65, 147.63, 147.61, 147.23, 147.05, 146.92, 144.48, 144.22, 143.53, 143.50, 143.07, 138.15, 137.47, 137.37, 137.29, 137.27, 137.18, 134.07, 133.40, 133.38, 132.99, 132.97, 132.86, 132.84, 132.11, 131.37, 131.09, 130.29, 129.86, 129.77, 129.61, 129.55, 129.48, 129.03, 129.02, 129.01, 128.82, 128.43, 128.37, 128.21, 128.01, 127.99, 127.95, 127.61, 127.40, 127.38, 124.31, 123.02, 123.87, 122.62, 120.13, 119.31, 114.77, 114.71, 114.66, 112.91, 112.90, 112.86, 112.79, 112.74, 66.40, 66.09, 55.80, 55.52, 55.44, 40.65, 40.64, 40.55, 40.47, 40.45, 40.40, 36.78, 36.68, 34.54, 33.30, 32.70, 32.11, 32.08, 30.87, 30.54, 30.49, 29.84, 29.81, 29.75, 29.73, 29.68, 29.59, 29.53, 29.51, 29.46, 28.80, 28.38, 27.89, 27.82, 27.75, 27.00, 26.53, 26.17, 23.37, 22.97, 22.94, 22.89, 22.87, 22.82, 19.77, 19.38, 14.32, 14.30, 13.91, 13.87, 11.59, 10.53, 10.36. MS (MALDI-TOF): Calcd. for $C_{71}H_{86}N_8OS_5$ $[M]^+$: 1226.552, Found: 1226.547.

9,9'-((12,13-bis(2-ethylhexyl)-3,9-diundecyl-12,13-dihydro-[1,2,5]thiadiazolo[3,4-*e*]thieno[2'',3'':4',5']thieno[2',3':4,5]pyrrolo[3,2-*g*]thieno[2',3':4,5]thieno[3,2-*b*]indole-2,10-diyl)bis(methanylylidene))bis(9*H*-indeno[1,2-*b*]pyrazine-2,3-dicarbonitrile) (IPC-BEH-IPC):

IPC-BEH-IPC was obtained during synthesis of compound **3** (Yield: 19%). ^{1}H NMR (400 MHz, $CDCl_3$) δ (ppm): 9.01-8.96, 8.26 & 7.97-7.94 (dd, d, & m, 2H), 8.74, 8.71, 8.29 & 8.20 (s, 2H), 8.17, 7.90-7.88 & 7.79-7.76 (d, m & m, 2H), 7.72-7.67 & 7.63-7.59 (t & t, 2H), 7.57-7.53 & 7.45-7.41 (t & t, 2H), 5.07-4.92 & 4.85-4.75 (m & m, 4H), 3.28-3.23 (m, 4H), 2.22-2.19 (m, 2H), 2.08-1.97 (m, 4H), 1.64-1.55 (m, 4H), 1.53-1.46 (m, 4H), 1.43-1.25 (m, 26H), 1.17-1.07 (m, 10H), 0.91-0.76 (m, 16H), 0.65-0.48 (m, 6H).^{13}C NMR (100 MHz, $CDCl_3$) δ (ppm): 154.61, 153.29, 152.70 152.68, 151.66, 150.38, 149.79, 149.76, 147.97, 147.95, 147.77, 147.73, 147.38, 147.37, 144.75, 144.73, 144.68, 144.53, 143.10, 142.88, 137.74, 134.19, 133.88, 132.75, 132.58, 131.60, 130.04, 129.56, 128.67, 128.43, 128.24, 128.10, 128.03, 127.75, 124.36, 124.03, 123.91, 123.89, 123.55, 122.66,, 122.58, 120.20, 119.70, 119.67, 119.42, 119.21, 114.81, 114.78, 114.66, 114.32, 114.30, 113.01, 112.97, 112.95, 77.43, 55.67, 55.42, 40.85, 40.69, 40.57, 32.12, 30.90, 30.55, 30.12, 30.02, 29.95, 29.90, 29.86, 29.83, 29.78, 29.76, 29.75, 29.70, 29.63, 29.58, 29.55, 29.55, 29.25, 27.65, 23.31, 23.06, 22.97, 22.91, 22.83, 22.79, 14.35, 13.96, 13.91, 13.88, 10.91, 10.36. MS (MALDI-TOF): Calcd. for $C_{84}H_{90}N_{12}S_5$ $[M]^+$: 1426.601, Found: 1426.758.

Synthesis of (*E*)-9-((10-((*Z*)-(1-(dicyanomethylene)-5,6-difluoro-3-oxo-1*H*-inden-2(3*H*)-ylidene)methyl)-12,13-bis(2-ethylhexyl)-3,9-diundecyl-12,13-dihydro-[1,2,5]thiadiazolo[3,4-*e*]thieno[2'',3'':4',5']thieno[2',3':4,5]pyrrolo[3,2-*g*]thieno[2',3':4,5]thieno[3,2-*b*]indol-2-yl)methylene)-9*H*-indeno[1,2-*b*]pyrazine-2,3-dicarbonitrile (IPC-BEH-IC2F):

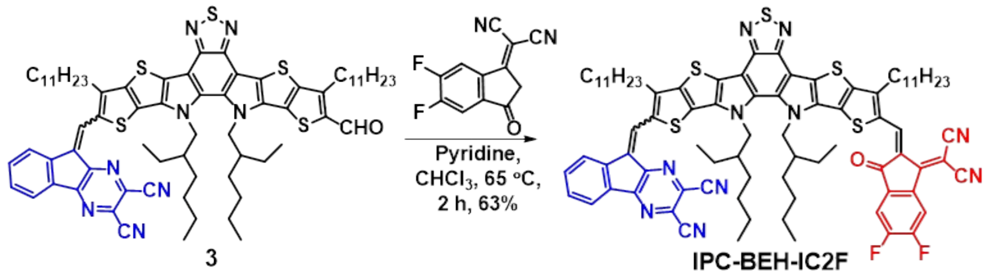

In a two-neck round-bottom flask, **3** (270 mg, 0.2199 mmol) and IC2F (126.53 mg, 0.5497 mmol) were added, and 15 mL of anhydrous chloroform was added under the argon atmosphere.

After that, pyridine (0.2 mL) was added and stirred at 65 °C for 2 h and the reaction mixture cooled to room temperature. After removing the solvent, the crude product was purified by silica gel column chromatography with chloroform as eluent. Dark blue color solid was obtained as product (Yield: 63%). ^{1}H NMR (400 MHz, $CDCl_3$) δ (ppm): 9.12 & 9.11 (s, 1H), 8.98 & 8.22 (d, J=4 & J=4 Hz, 1H), 8.73 & 8.35 (s, 1H), 8.54-8.50 (m, 1H), 8.32 & 7.96 (d, 1H), 7.80-7.68 (m, 2H) 7.58 (t, 1H), 4.87-4.74 (m, 4H), 3.28-3.20 (m, 4H), 2.19-12 (br, 2H), 2.01-1.94 (m, 2H), 1.91-1.84 (m, 2H), 1.55-1.40 (m, 6H), 1.37-1.26 (m, 30 H), 1.19-0.91 (m, 12 H), 0.88-0.76 (m, 12 H), 0.74-0.65 (m, 3H), 0.63-0.55 (m, 3H). ^{13}C NMR (100 MHz, $CDCl_3$) δ (ppm): 189.50, 186.33, 159.15, 159.09, 155.88, 154.28, 154.16, 153.60, 153.14, 151.31, 150.66, 147.80, 147.78, 147.71, 147.50, 146.97, 145.09, 144.79, 143.14, 138.24, 137.57, 137.53, 136.86, 136.79, 136.61, 136.57, 136.44, 135.71, 135.56, 135.47, 135.41, 134.73, 134.67, 134.51, 133.31, 133.24, 133.12, 131.78, 131.13, 130.56, 129.71, 128.62, 128.57, 128.44, 128.09, 127.90, 127.21, 124.05, 120.82, 119.85, 119.57, 119.53, 115.27, 115.21, 114.99, 114.88, 114.77, 114.72, 113.91, 113.04, 112.99, 112.78, 112.65, 112.60, 101.59, 77.43, 68.50, 55.89, 40.79, 40.50, 32.14, 31.45, 30.90, 30.15, 30.06, 29.99, 29.93, 29.87, 29.84, 29.74, 29.68, 29.56, 29.07, 28.24, 28.09, 27.66, 27.61, 23.69, 23.63, 23.36, 23.05, 23.00, 22.89, 14.33, 14.01, 13.99, 13.88, 10.60, 10.57, 10.50, ^{19}F NMR (564 MHz, $CDCl_3$) δ (ppm): -123.13 (br, 1F), 124.41(br, 1F). MS (MALDI-TOF): Calcd. for $C_{83}H_{88}F_2N_{10}OS_5$ $[M]^+$: 1438.571, Found: 1438.655.

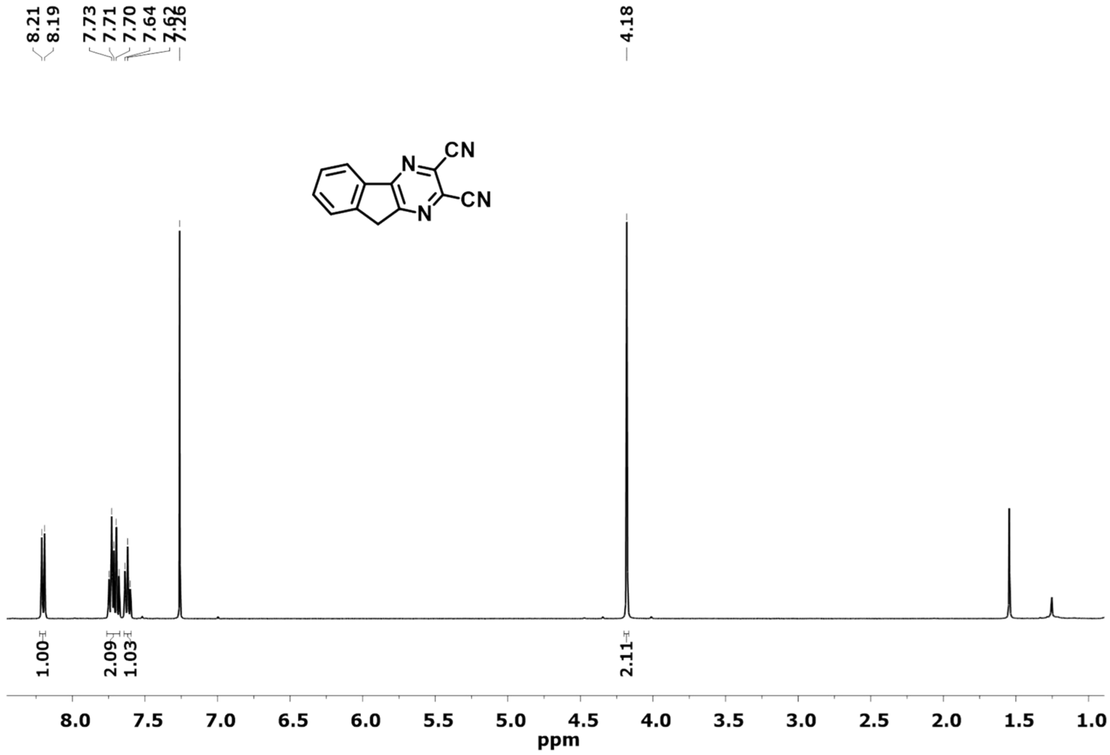

Figure S1. ^{1}H NMR spectrum of the **IPC.**

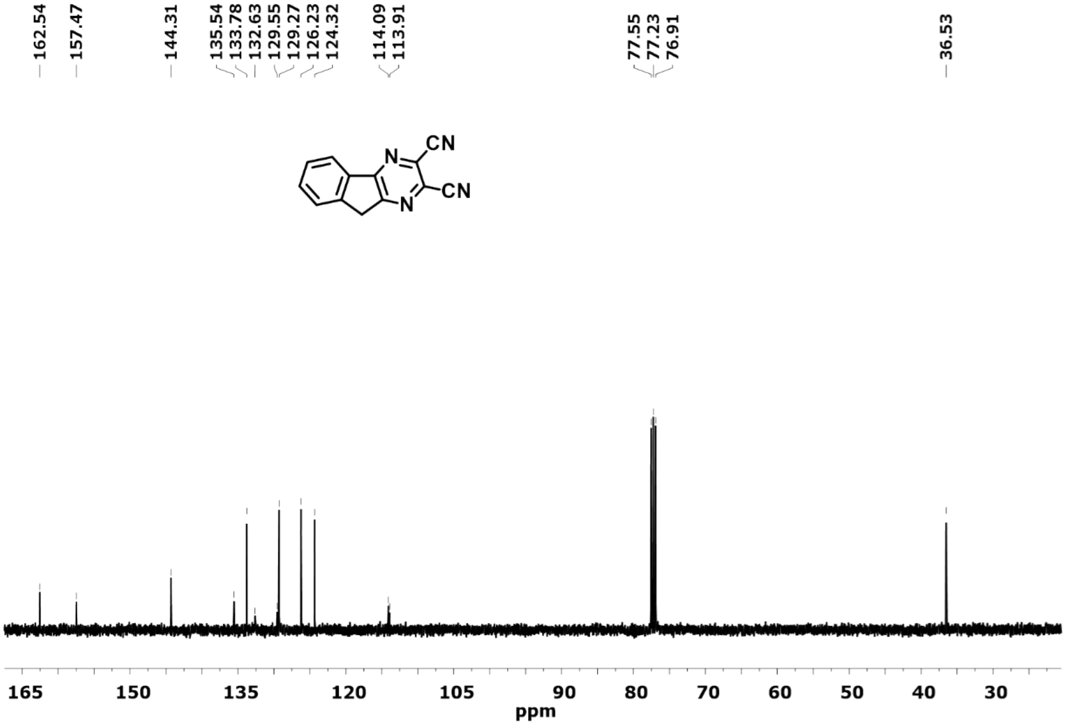

Figure S2. ^{13}C NMR spectrum of the **IPC.**

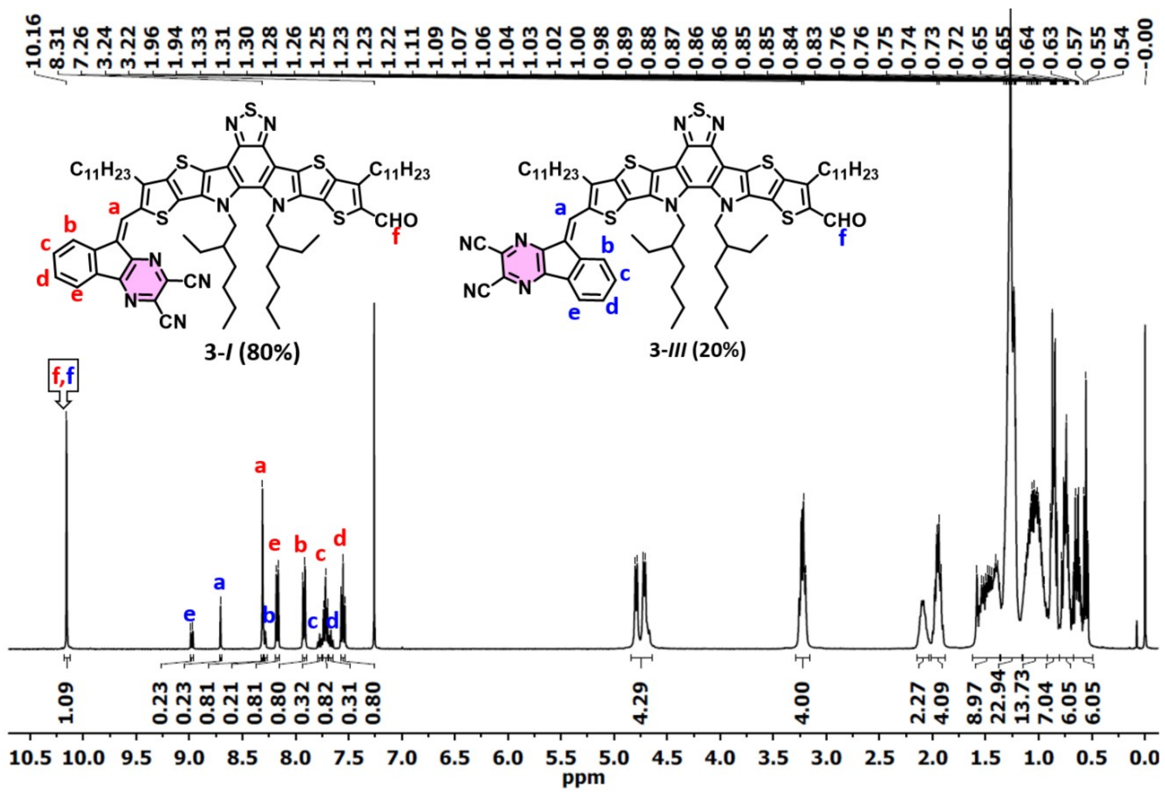

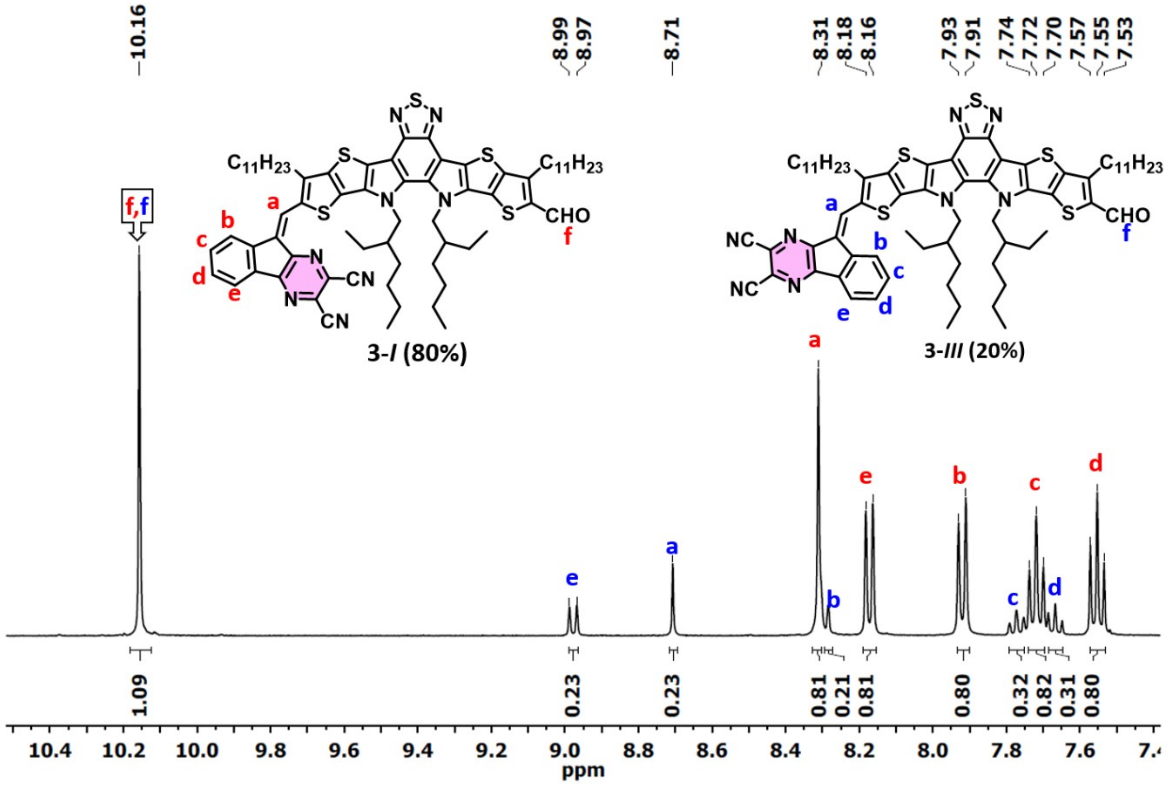

Figure S3. ^{1}H NMR spectrum of **3.**

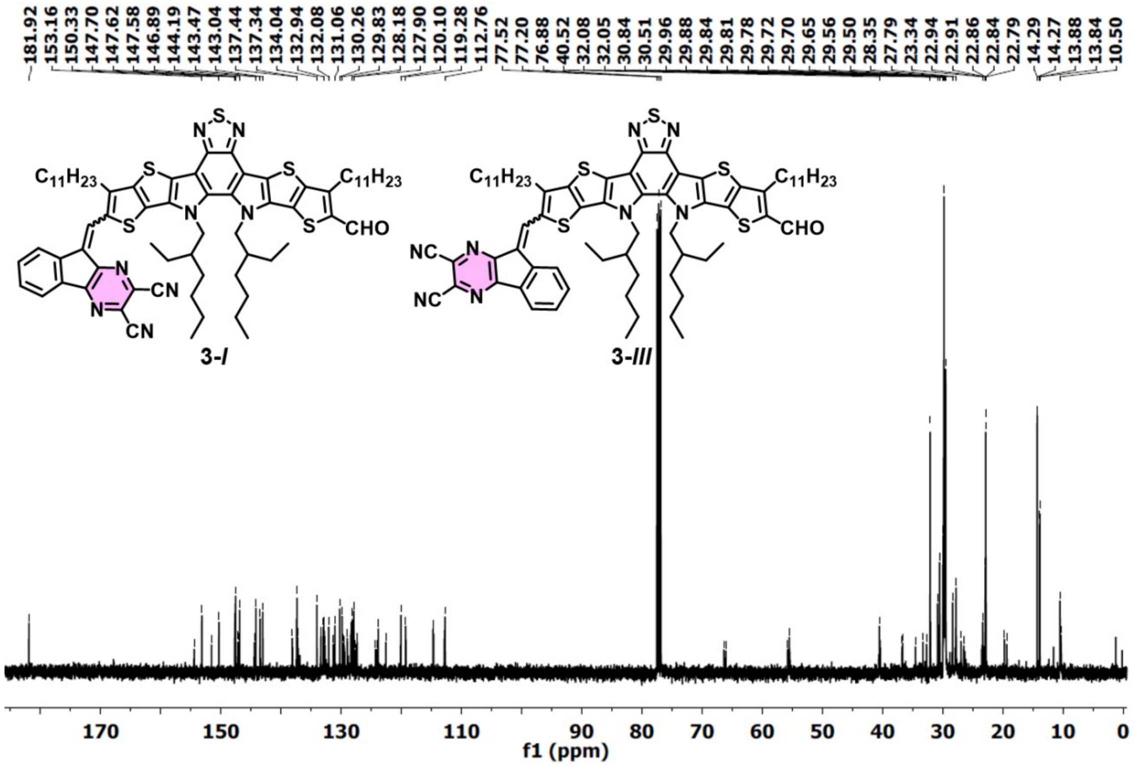

Figure S4. ^{13}C NMR spectrum of **3.**

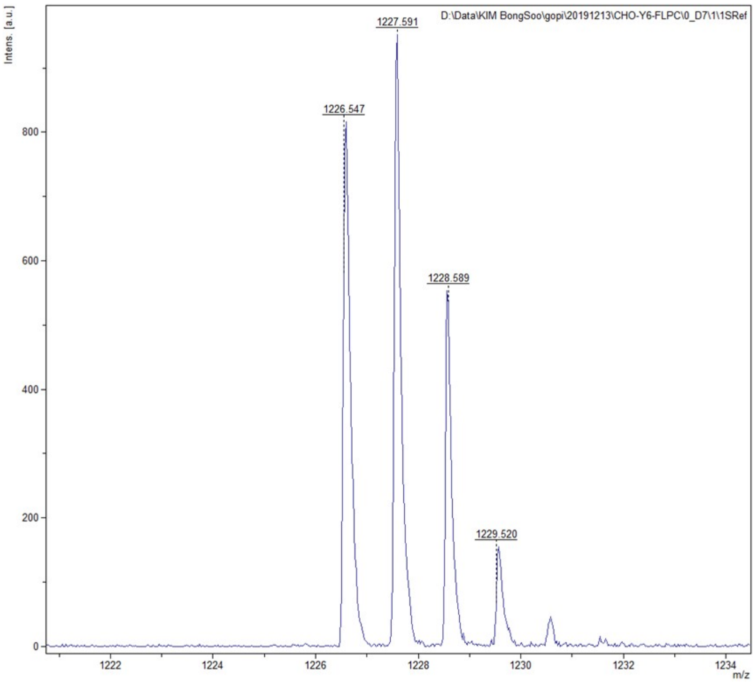

Figure S5. MS (MALDI-TOF) of **3.**

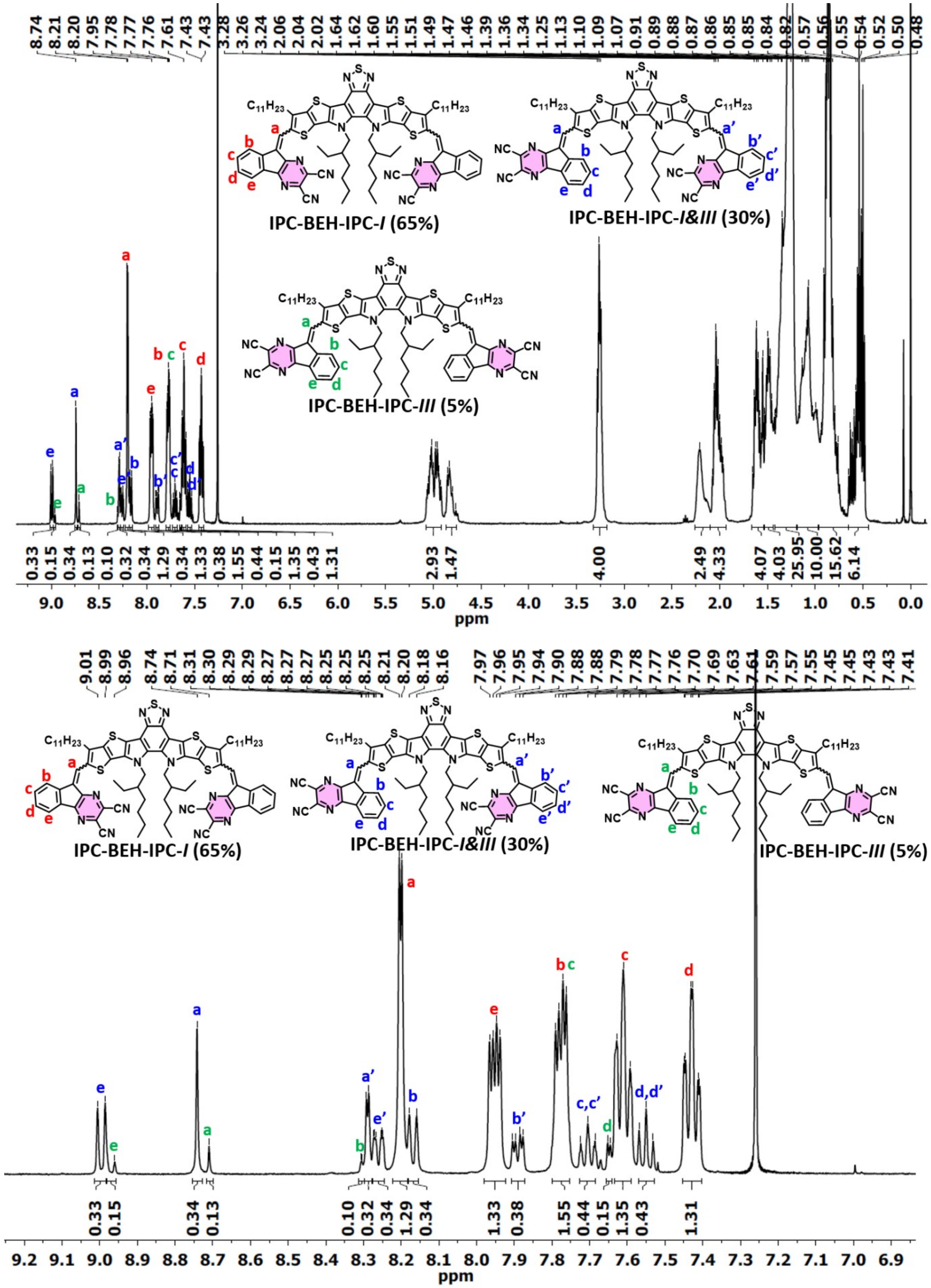

Figure S6. ^{1}H NMR spectrum of the **IPC-BEH-IPC.**

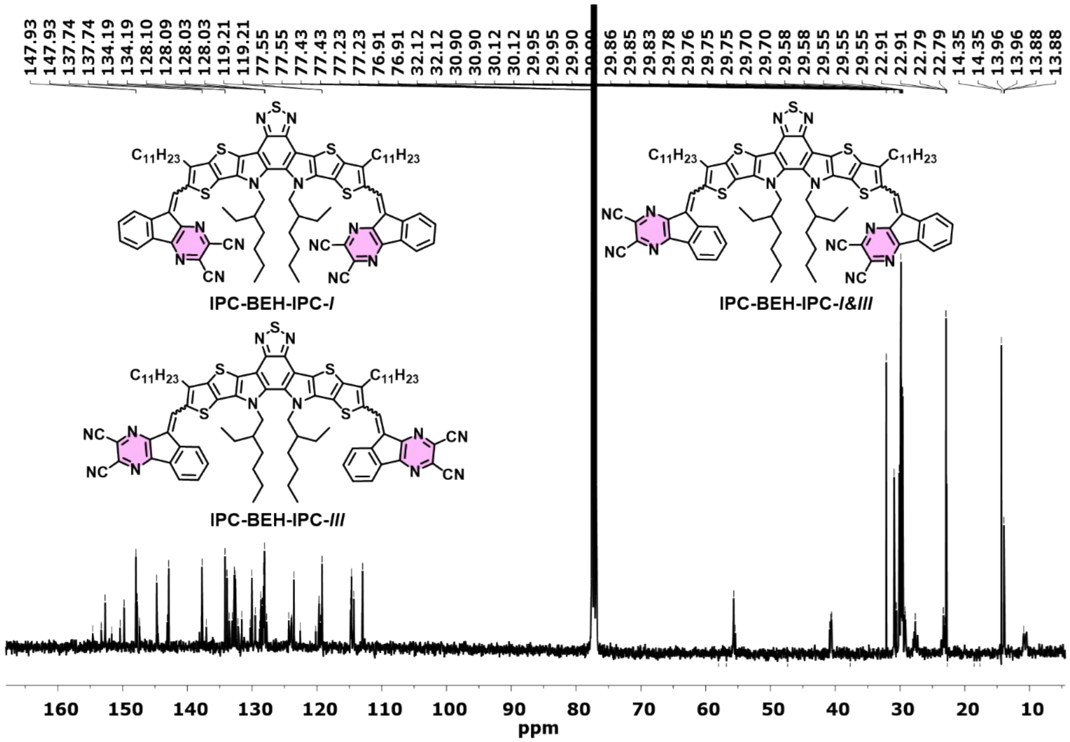

Figure S7. ^{13}C NMR spectrum of the **IPC-BEH-IPC.**

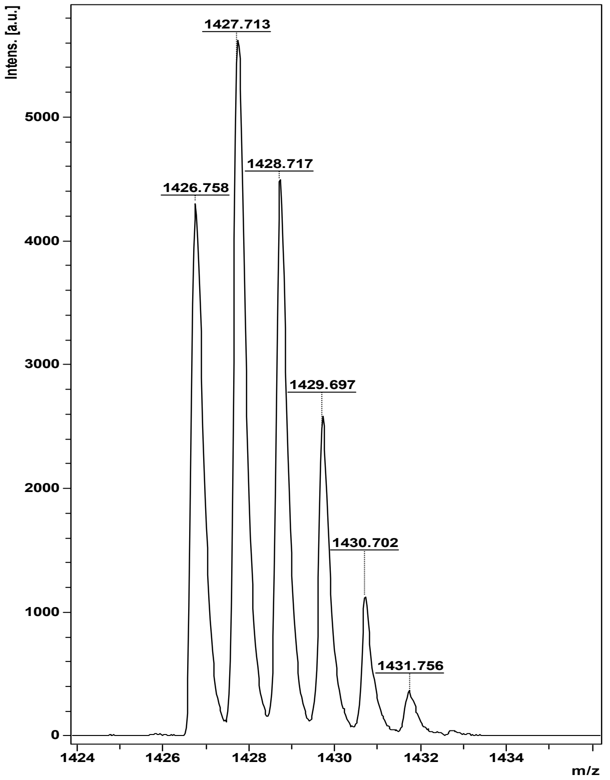

Figure S8. MS (MALDI-TOF) of the **IPC-BEH-IPC.**

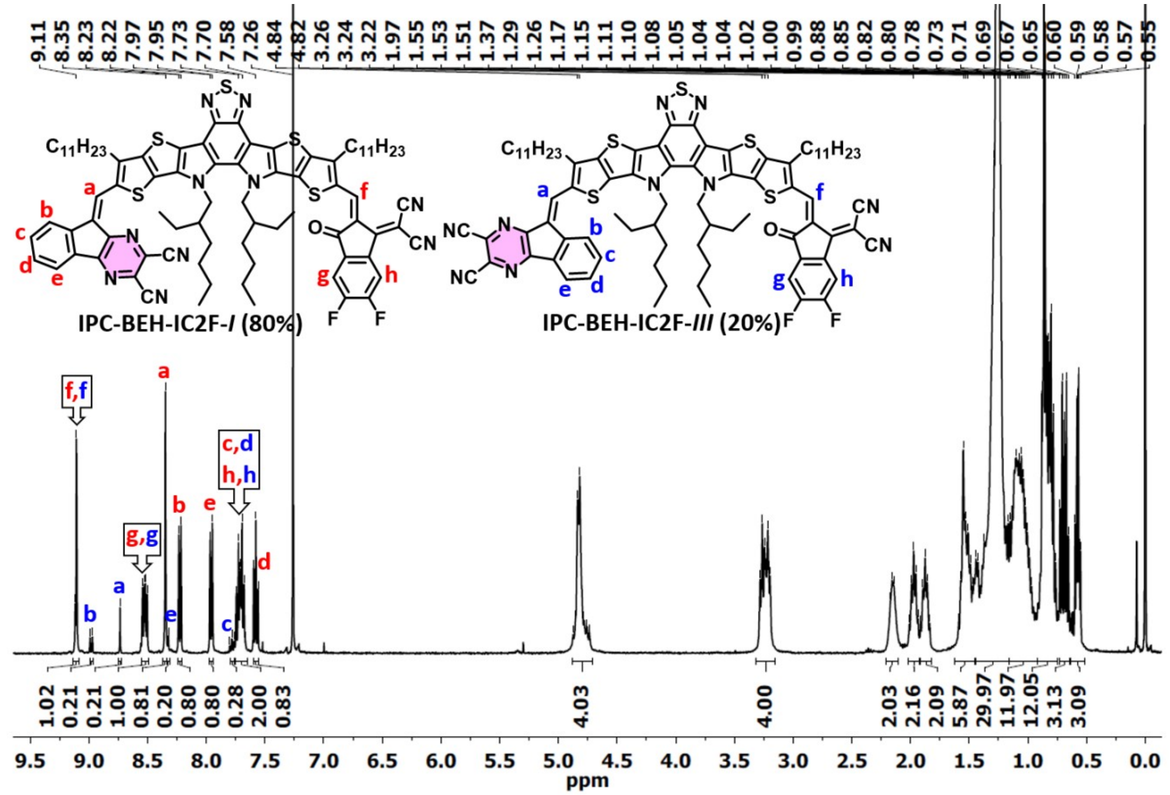

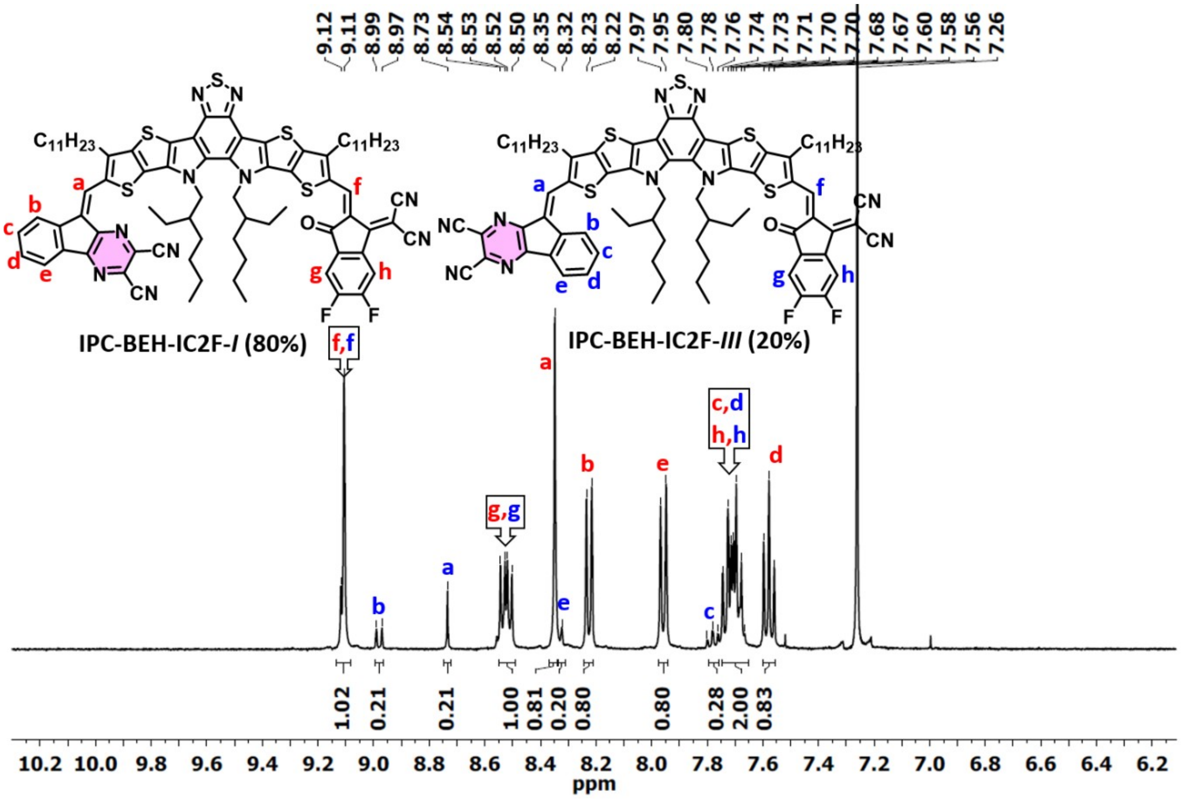

Figure S9. ^{1}H NMR spectrum of the **IPC-BEH-IC2F.**

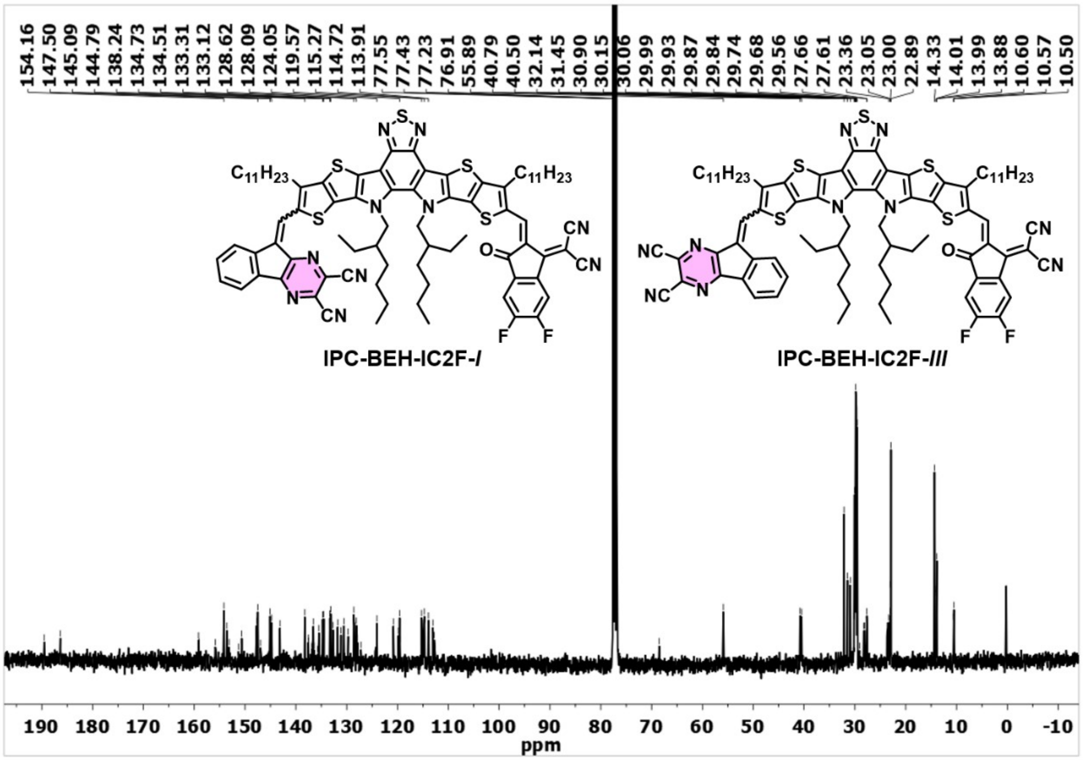

Figure S10. ^{13}C NMR spectrum of the **IPC-BEH-IC2F.**

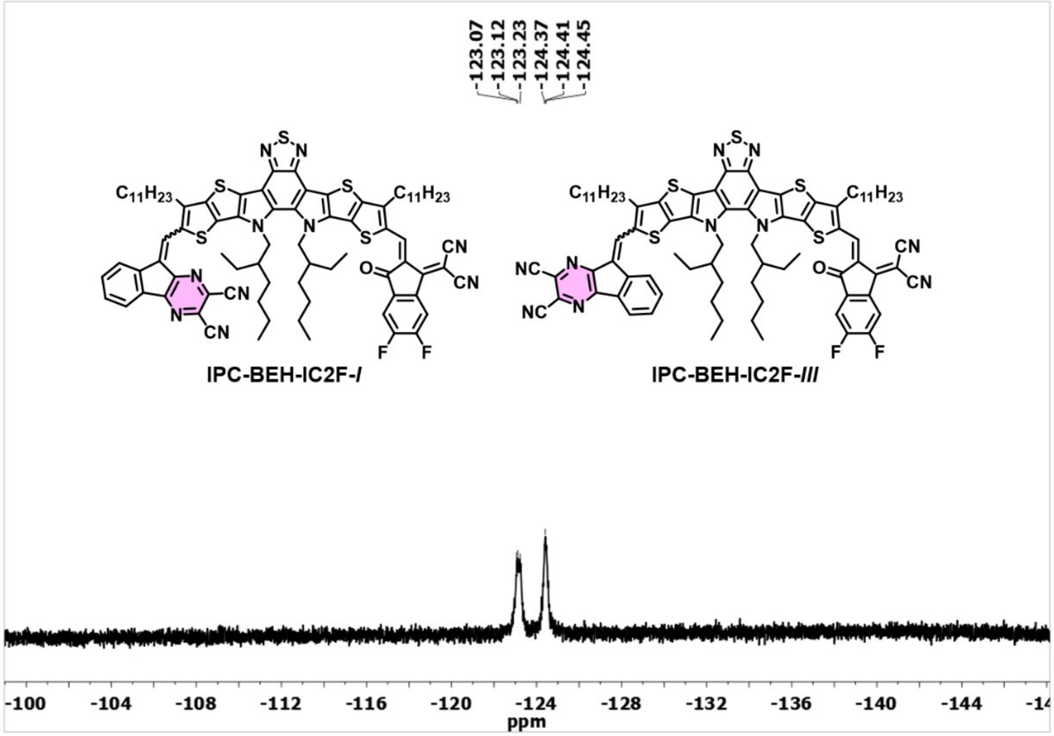

Figure S11. ^{19}F NMR spectrum of the **IPC-BEH-IC2F.**

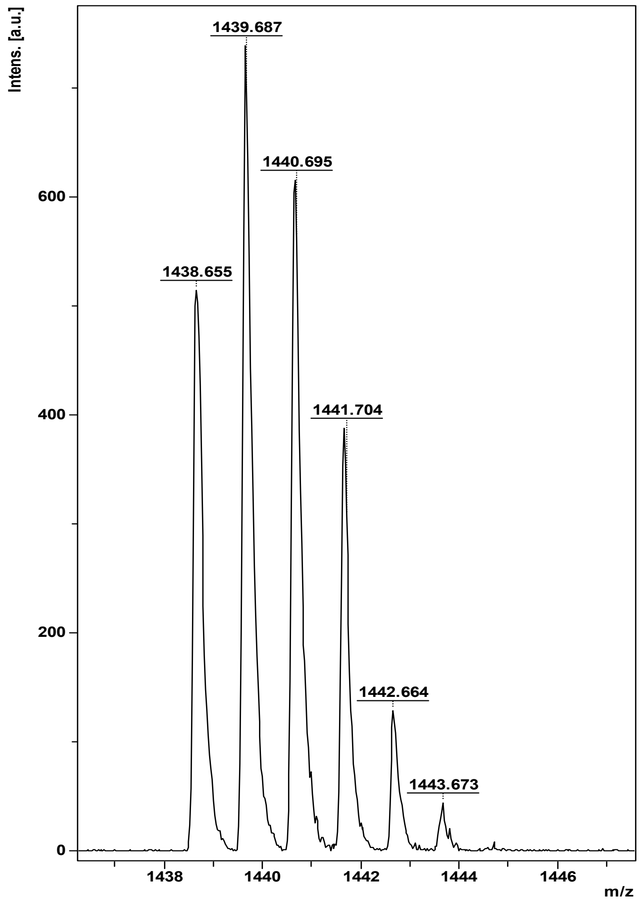

Figure S12. MS (MALDI-TOF) of the **IPC-BEH-IC2F.**

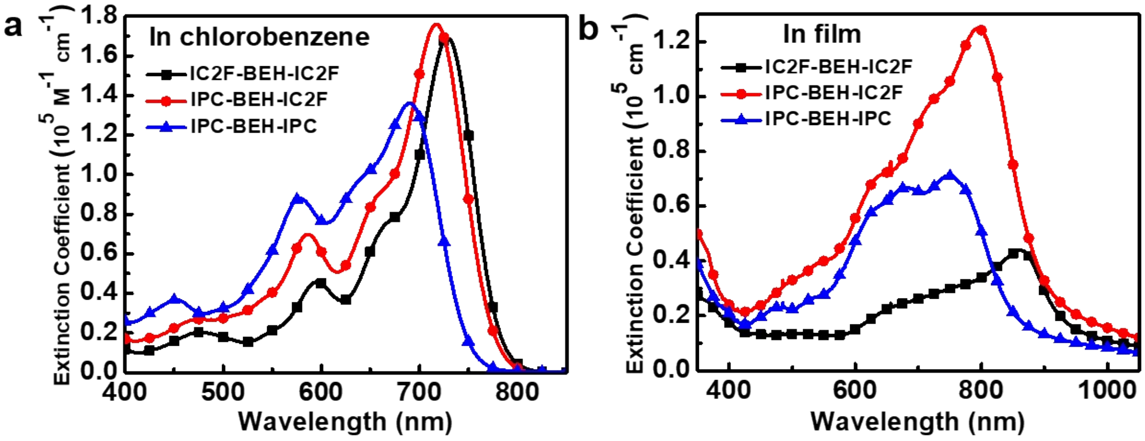

Figure S13. UV-vis absorption spectra of the NFAs in (a) chlorobenzene solution and (b) film.

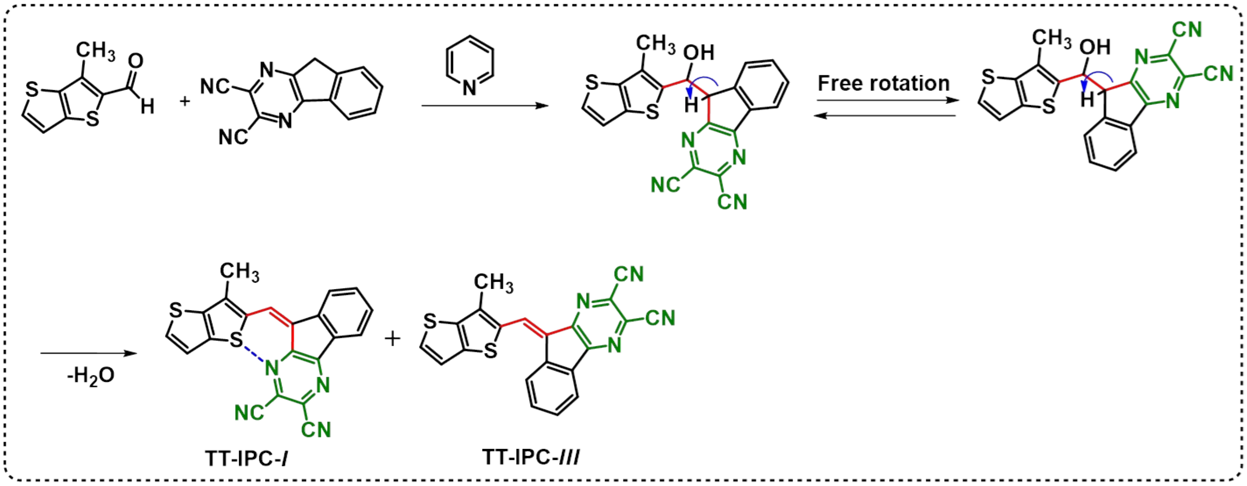

Figure S14. Proposed reaction mechanism for the formation of TT-IPC-*I* and TT-IPC-*III*.

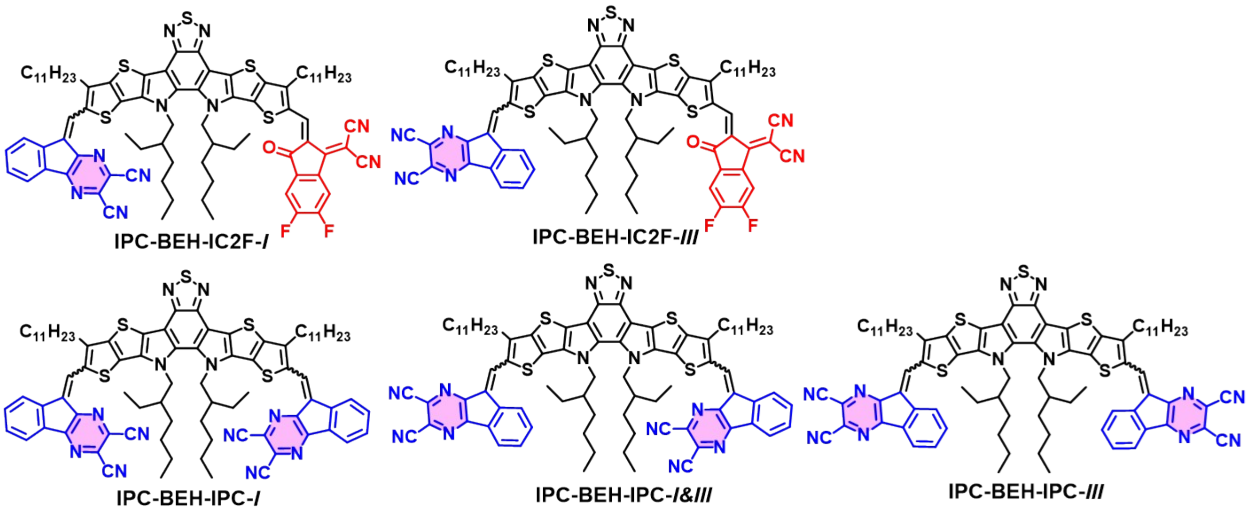

Figure S15. Possible isomers for IPC-BEH-IC2F and IPC-BEH-IPC.

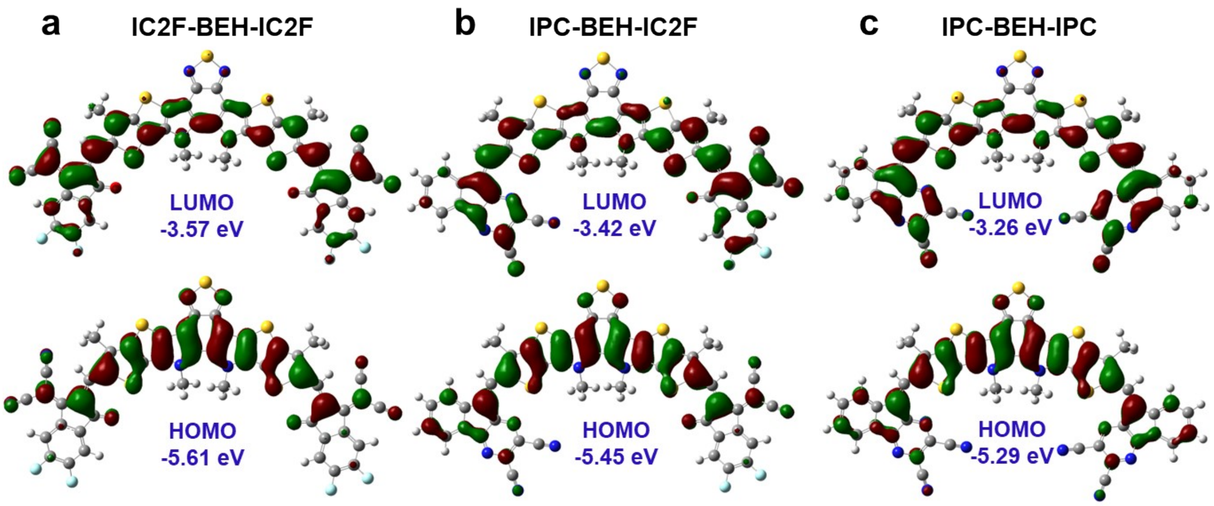

Figure S16. Surface plots of HOMO and LUMO levels of NFAs: (a) IC2F-BEH-IC2F, (b) IPC-BEH-IC2F, and (c) IPC-BEH-IPC.

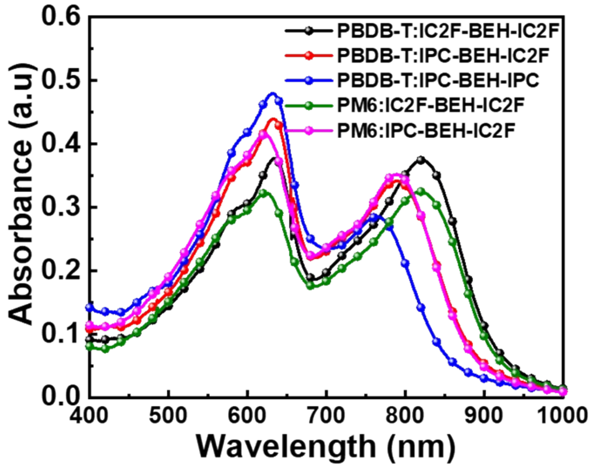

Figure S17. UV-vis absorption spectra of the polymer:NFA blend films.

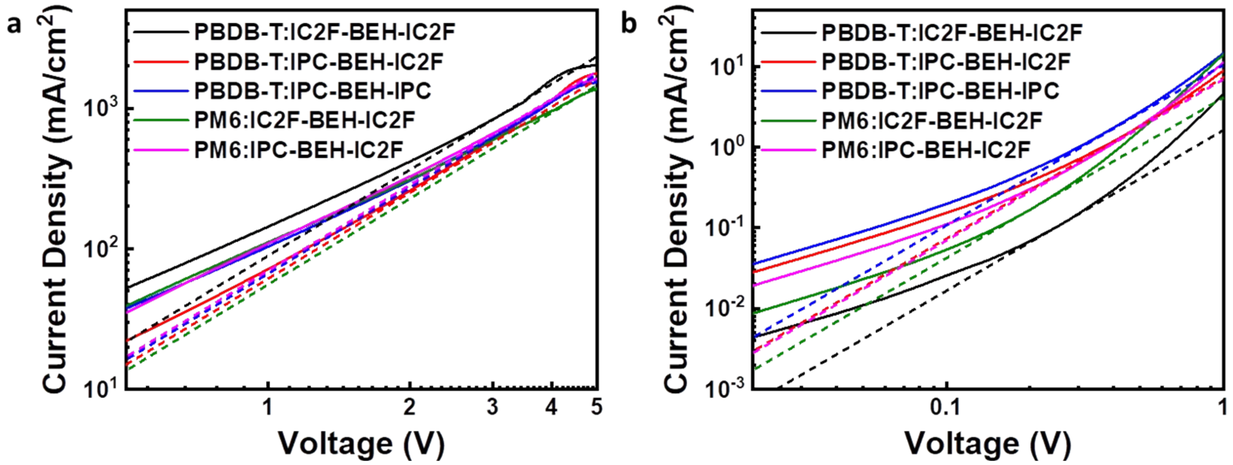

Figure S18. Space-charge limited current (SCLC) curves of (a) hole-only devices and (b) electron-only devices.

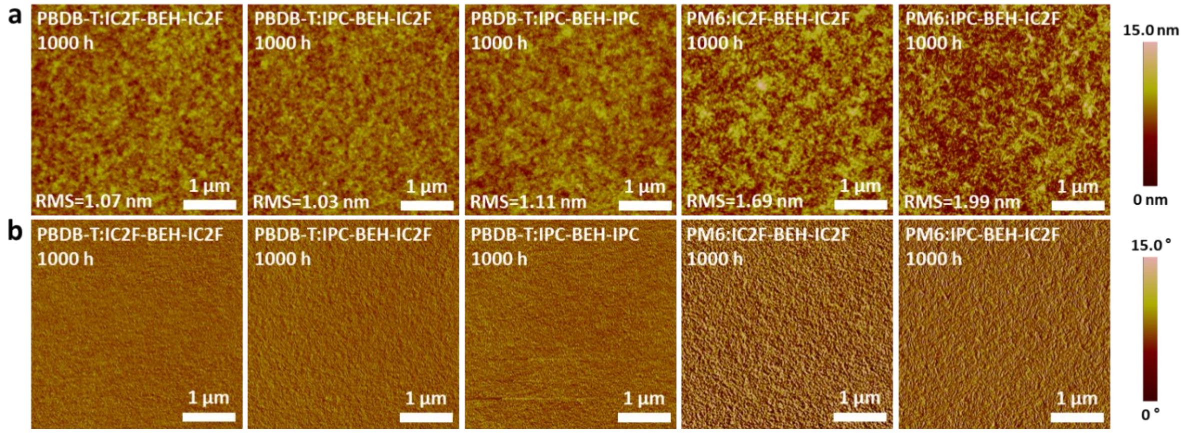

Figure S19. AFM (a) height images and (b) phase images of 1000 h-aged polymer:NFA films under ambient conditions.

Table S1. Measurement of the hole and electron mobilities.

Active layer	Hole Mobility ($cm^2V^{-1}s^{-1}$)	Electron Mobility ($cm^2V^{-1}s^{-1}$)
**PBDB-T:IC2F-BEH-IC2F**	$5.19(\pm 0.55) \times 10^{-4}$	$7.33(\pm 0.96) \times 10^{-6}$
**PBDB-T:IPC-BEH-IC2F**	$2.20(\pm 0.43) \times 10^{-4}$	$3.27(\pm 0.67) \times 10^{-5}$
**PBDB-T:IPC-BEH-IPC**	$2.57(\pm 0.39) \times 10^{-4}$	$4.94(\pm 0.65) \times 10^{-5}$
**PM6:IC2F-BEH-IC2F**	$2.82(\pm 0.46) \times 10^{-4}$	$1.81(\pm 0.24) \times 10^{-5}$
**PM6:IPC-BEH-IC2F**	$3.22(\pm 0.50) \times 10^{-4}$	$3.13(\pm 0.19) \times 10^{-5}$

Table S2. Summary of GIWAXS parameters of neat films of polymers and NFAs.

Sample	Lamellar spacing from (100) in the IP direction						*π-π* spacing from (010) in the OOP direction					
	q (Å^{-1})	*d* (Å)	Height (a.u.)	Area (a.u.)	FWHM (Å^{-1})	L_C (nm)	*q* (Å^{-1})	*d* (Å)	Height (a.u.)	Area (a.u.)	FWHM (Å^{-1})	L_C (nm)
**PBDB-T**	0.288	21.826	1328.05	106.60	0.051	11.066	1.701	3.695	413.91	158.94	0.245	2.312
**PM6**	0.287	21.898	928.39	59.03	0.040	13.967	1.694	3.709	185.32	65.61	0.237	2.388
**IC2F-BEH-IC2F**	0.269	23.369	722.48	92.00	0.093	6.089	1.760	3.570	88.88	14.41	0.152	3.714
**IPC-BEH-IC2F**	0.222	28.320	232.42	5.40	0.021	26.730	1.757	3.576	703.23	178.84	0.122	4.622
**IPC-BEH-IPC**	0.263	23.934	304.25	57.03	0.129	4.388	1.727	3.638	1194.88	503.88	0.270	2.097

Table S3. Summary of GIWAXS parameters of polymer:NFA blend films.

Sample	Lamellar spacing from (100) in the IP direction						*π-π* spacing from (010) in the OOP direction					
	q (Å^{-1})	*d* (Å)	Height (a.u.)	Area (a.u.)	FWHM (Å^{-1})	L_C (nm)	*q* (Å^{-1})	*d* (Å)	Height (a.u.)	Area (a.u.)	FWHM (Å^{-1})	L_C (nm)
**PBDB-T:IC2F-BEH-IC2F**	0.286	21.965	5410.24	378.79	0.048	11.738	1.736	3.619	1260.45	386.63	0.203	2.785
**PBDB-T:IPC-BEH-IC2F**	0.287	21.864	4346.52	294.97	0.043	13.051	1.734	3.624	1253.77	424.19	0.228	2.475
**PBDB-T:IPC-BEH-IPC**	0.284	22.137	3229.82	210.35	0.042	13.598	1.721	3.652	1049.05	381.10	0.273	2.074

**PM6:IC2F-BEH-IC2F**	0.288	21.797	1901.55	216.12	0.074	7.693	1.742	3.607	500.28	154.54	0.215	2.633
**PM6:IPC-BEH-IC2F**	0.289	21.731	2070.66	131.86	0.040	13.946	1.727	3.637	744.84	265.75	0.227	2.490

Table S4. Summary of GIWAXS parameters of 1000 h-aged polymer:NFA blend films.

**Sample**	**Lamellar spacing from (100) in the IP direction**						***π-π* spacing from (010) in the OOP direction**					
	q **(Å^{-1})**	***d*** **(Å)**	**Height (a.u.)**	**Area (a.u.)**	**FWHM (Å^{-1})**	**L_C (nm)**	***q*** **(Å^{-1})**	***d*** **(Å)**	**Height (a.u.)**	**Area (a.u.)**	**FWHM (Å^{-1})**	**L_C (nm)**
**PBDB-T:IC2F-BEH-IC2F**	0.285	22.085	2264.1	212.5	0.066	8.575	1.731	3.630	789.5	261.5	0.211	2.682
**PBDB-T:IPC-BEH-IC2F**	0.288	21.785	1880.2	153.8	0.055	10.280	1.732	3.627	863.3	288.6	0.228	2.477
**PBDB-T:IPC-BEH-IPC**	0.286	21.994	1786.0	151.0	0.055	10.315	1.714	3.666	774.8	321.0	0.271	2.090
**PM6:IC2F-BEH-IC2F**	0.287	21.889	1980.3	223.5	0.073	7.712	1.738	3.614	403.3	113.2	0.209	2.704
**PM6:IPC-BEH-IC2F**	0.288	21.802	1779.3	131.1	0.047	12.018	1.725	3.642	705.4	252.3	0.237	2.388

References:

S1. M. H. F. Overwijk et al., *J. Vac. Sci. Technol. B*, 1993, **11**, 2021-2024.
